# The Coordinated Activities of nAChR and Wnt Signaling Regulate Intestinal Stem Cell Function in Mice

**DOI:** 10.3390/ijms19030738

**Published:** 2018-03-05

**Authors:** Toshio Takahashi, Akira Shiraishi, Jun Murata

**Affiliations:** Suntory Foundation for Life Sciences, Bioorganic Research Institute, Kyoto 619-0284, Japan; shiraishi@sunbor.or.jp (A.S.); murata-j@sunbor.or.jp (J.M.)

**Keywords:** nAChRs, non-neuronal ACh, organoid, Wnt5a, intestinal stem cells

## Abstract

Cholinergic signaling, which modulates cell activities via nicotinic and muscarinic acetylcholine receptors (n- and mAChRs) in response to internal or external stimuli, has been demonstrated in mammalian non-neuronal cells that synthesize acetylcholine (ACh). One of the major pathways of excitatory transmission in the enteric nervous system (ENS) is mediated by cholinergic transmission, with the transmitter ACh producing excitatory potentials in postsynaptic effector cells. In addition to ACh-synthesizing and ACh-metabolizing elements in the ENS, the presence of non-neuronal ACh machinery has been reported in epithelial cells of the small and large intestines of rats and humans. However, little is known about how non-neuronal ACh controls physiological function in the intestine. Here, experiments using crypt–villus organoids that lack nerve and immune cells in culture suggest that endogenous ACh is synthesized in the intestinal epithelium to drive organoid growth and differentiation through activation of nAChRs. Treatment of organoids with nicotine enhanced cell growth and the expression of marker genes for stem and epithelial cells. On the other hand, the nAChR antagonist mecamylamine strongly inhibited the growth and differentiation of organoids, suggesting the involvement of nAChRs in the regulation of proliferation and differentiation of Lgr5-positive stem cells. More specifically, RNA sequencing analysis revealed that *Wnt5a* expression was dramatically upregulated after nicotine treatment, and Wnt5a rescued organoid growth and differentiation in response to mecamylamine. Taken together, our results indicate that coordinated activities of nAChR and Wnt signaling maintain Lgr5-positive stem cell activity and balanced differentiation. Furthermore, we could clearly separate the two groups, neuronal ACh in the ENS and non-neuronal ACh in the intestinal epithelium. Dysfunction of the non-neuronal cholinergic system is involved in the pathogenesis of disease. The data will increase our understanding of the cholinergic properties of non-neuronal cells and lead to optimization of drug therapy.

## 1. Introduction

The mouse small intestinal epithelium is composed of crypt–villus units that are in a continuous state of homeostatic cell turnover. Thus, the tissue is a pivotal model for studying tissue renewal. Six lineages of differentiated epithelial cells have been described in the crypt–villus unit, and the most populous of these are goblet cells, enteroendocrine cells, Paneth cells, and enterocytes [1]. These four cell types are continuously produced by intestinal stem cells (ISCs) located near the base of the intestinal crypts. Genetic lineage tracing studies have identified distinct ISC populations, including crypt base columnar cells that are marked by *Lgr5* expression [2]. *Lgr5* is a Wnt target gene, and the protein is a leucine-rich repeat-containing G-protein-coupled receptor whose ligand is an R-spondin [3]. Recently, Sato and co-workers presented a novel method that allows long-term culture of isolated intestinal crypts or *Lgr5*-positive ISCs [4]. Supplemented with the appropriate growth factor cocktail (epidermal growth factor (EGF), Noggin, and R-spondin-1) and cultured in a three-dimensional extracellular matrix, the ISCs are capable of developing into organoids. The mouse intestinal organoid grows as a single-layered epithelium organized into domains such that it resembles the in vivo intestinal crypt–villus architecture, comprising the different cell types of the intestine (enterocytes, goblet cells, Paneth cells, enteroendocrine cells, and stem cells) and surrounding a cystic lumen [4]. Furthermore, single Lgr5-positive ISCs can also initiate the formation of these crypt–villus organoids that retain hallmarks of the in vivo epithelium [4].

Although the role of ACh in neurotransmission within the nervous system is well understood, less is known about the physiological roles of non-neuronal ACh. The most detailed analysis of cellular function of non-neuronal ACh has been evaluated for keratinocytes of the human skin [5]. Morris [6] first described non-neuronal ACh synthesis in the placenta in 1966, and subsequently ACh and/or the synthesizing enzyme choline acetyltransferase were detected in epithelia lining airways, the alimentary tract, the urogenital tract, and the epidermis [7]. Thus, ACh likely plays a role in mediating and regulating various critical cellular processes such as cell division, differentiation, and establishment of cell–cell contacts. ACh may have paracrine and autocrine functions as well [8,9]. Collectively, in addition to a classical role in the nervous system, an emerging concept is that ACh may be a universal regulator of biological systems [10].

Development of the small intestine and homeostasis in the adult intestine requires Wnt signaling [11,12,13,14]. The multiple Wnt ligands in vertebrates signal through two distinct mechanisms: the canonical Wnt/β-catenin and the non-canonical Wnt pathways. Canonical Wnt signaling is important for maintaining stem cell fate and induces proliferation of stem cells with activation of β-catenin [15,16]. Moreover, Wnt signaling also enhances the differentiation of Paneth cells, which are in direct contact with ISCs [17]. One of the non-canonical Wnt ligands is Wnt5a, which is expressed in the gut mesenchyme during mouse development [18,19]. *Wnt5a* mutant mice display a dramatic shortening of the small intestine accompanied by an aberrant bifurcation of the midgut [14]. In adult mice, Wnt5a-positive mesenchymal cells support crypt structure formation in damaged areas [20].

We investigated the function of non-neuronal ACh using crypt–villus organoids lacking nerve, immune, and mesenchymal cells. We found that non-neuronal ACh enhanced the growth and differentiation of crypt–villus organoids, and was involved in both the proliferation and differentiation of Lgr5-positive stem cells in the mouse intestine via nicotinic AChRs (nAChRs). Furthermore, we found that the non-canonical Wnt5a pathway functioned downstream of nAChR signaling to coordinate the nicotinic effect. These data demonstrate a coordinating regulatory mechanism that maintains homeostasis of intestinal epithelial cell growth and differentiation via nAChRs in mice.

## 2. Results

### 2.1. Organoids Contain Non-Neuronal nAChRs

**Figure 1:**
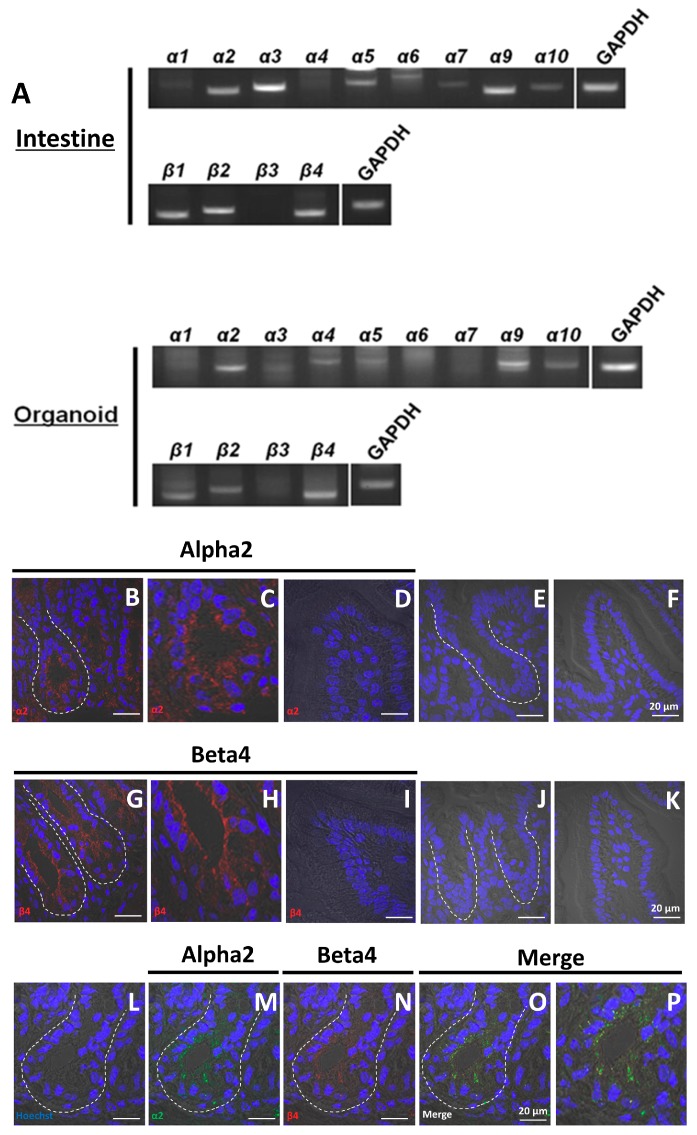
Localization of nAChR subunits in the mouse small intestine and organoids. (**A**) RT-PCR analysis of the expression of nAChR subunits in intestine and cultured organoids (passage 5); (**B**,**D**) visualization of α2 (red) in crypts and villus; (**E**,**F**) control sections labeled with secondary antibody [Alexa Fluor 546 donkey anti-(rabbit IgG)] in the absence of primary antibody; (**G**,**I**) visualization of β4 (red) in crypts and villus; (**J**,**K**) control sections labeled with secondary antibody [Alexa Fluor 568 rabbit anti-(goat IgG)] in the absence of primary antibody; (**L**–**O**) co-localization of α2 and β4 in crypts; (**M**) visualization of α2 (green) in crypts; (**N**) visualization of β4 (red) in crypts; (**O**) merged visualization of (**L**), (**M**), and (**N**). (**C**,**H**,**P**) Enlargement of (**B**), (**G**), and (**O**). White dotted lines indicate the crypt region. In all panels, nuclei were stained with Hoechst 33342 (blue). Bars in (**B**–**P**) except for (**C**), (**H**), and (**P**) represent 20 μm.

Organoids derived from crypts are composed of ISCs and epithelial cells. Previously, we showed that a diverse array of nAChRs was expressed in organoids [21]. Thus, we examined the expression patterns of other nAChR subunits in the organoids in detail. RT-PCR analysis revealed that the expression patterns of α and β subunits in cultured organoids are generally consistent with that in intestine (Figure 1A). Although the expression of mRNA encoding the α3 subunit was observed in both tissues, *α3* expression in organoids was weaker than that in the intestine (Figure 1A). Overall, these data indicate that organoids largely retain the characteristic expression patterns of intestinal nAChRs.

nAChRs are cation-permeable ligand-gated ion channels, some of which are formed by heteropentameric αβ combinations of α (2–6) and β (2–4), and others (α7–α9) form homomeric receptors [22]. According to the expression pattern of nAChR subunits shown in Figure 1A, we hypothesized that active forms of nAChRs, such as α2/β2, α2/β4, α3/β2, α4/β2, and α9 exist in gut epithelium. To test this hypothesis, we carried out immunohistochemical staining using antibodies against α2, α3, α9, β2, and β4 nAChR subunits. As shown in Figure 1B,C,G,H, α2- and β4-specific antibodies (but not other α- and β-type antibodies) stained the cell membranes of epithelial cells in the crypt domain. However, we could not detect signals in villi (Figure 1D,I), and no immunostaining was observed in cells in the absence of primary antibody (Figure 1E,F,J,K). Furthermore, the α2 subunit present in the membrane showed co-localization with the β4 subunit (Figure 1L–P), indicating the presence of the α2/β4 nAChR subtype.

We next examined the effect of nicotine, a nicotinic receptor agonist, on organoid growth [23]. Organoid growth was enhanced by 10^−5^ M nicotine, but not by 10^−7^ M nicotine compared with the untreated control at day 4 (Figure S1A). However, nicotine appeared to have no effect at day 7 (Figure S1A). A time-course analysis revealed that the enhancement of organoid growth first reached significance at day 4 of 10^−5^ M nicotine treatment, in a manner that was not completely dose-dependent (Figure S1B). Endogenous ACh secreted from organoids [21] and exogenous nicotine compete for binding of nAChRs. As a result, the nicotinic effect on organoid growth is weak.

### 2.2. Inhibition of Organoid Growth and Differentiation by a nAChR Antagonist

**Figure 2:**
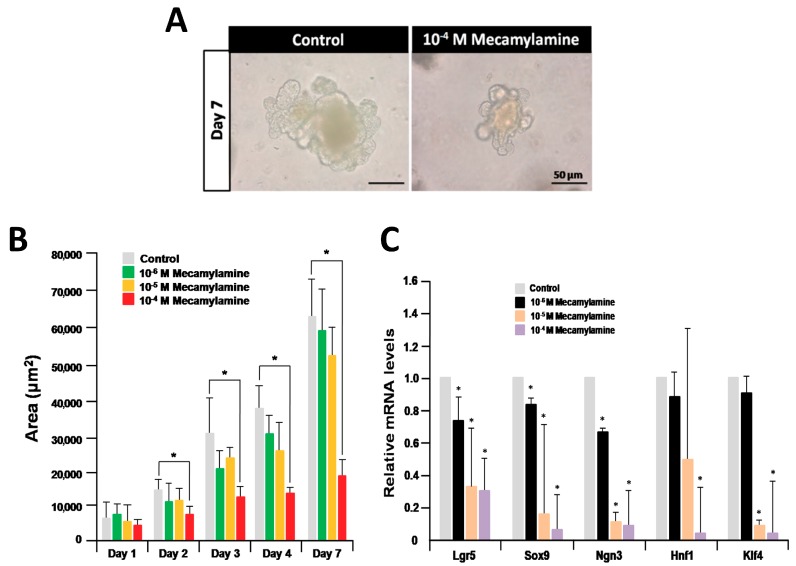
Effect of mecamylamine on organoid growth and differentiation. (**A**) Micrographs of organoids after treatment with 10^−4^ M mecamylamine for seven days of culture; (**B**) effect of mecamylamine on the size of cultured organoids. Each sample represents an average of three independent experiments. Error bars represent the standard deviation (SD) of the mean. An asterisk indicates a statistically significant difference from untreated control organoids (Mann–Whitney test, * *p* < 0.05). (**C**) Relative quantification of marker genes after treatment with mecamylamine for seven days. The results are based on three independent experiments and are expressed as mean values ± SD. The statistical significance was calculated with Student’s *t*-test (* *p* < 0.05 as compared with control).

nAChRs were recently implicated in the control of the growth and proliferation of mouse embryonic stem cells (ESCs) and induced pluripotent stem (iPS) cells [24,25]. As we only detected a mild effect of nicotine on organoid growth, we next examined the effect of mecamylamine, a nicotinic receptor antagonist, on cultured organoids [23]. Organoid growth was dramatically inhibited by treatment with 10^−4^ M mecamylamine compared with controls at day 7 (Figure 2A). Except for day 1, there was a statistically significant dose-dependent inhibition of organoid growth until day 7 (Figure 2B). Next, using quantitative RT-PCR we compared the expression levels of selected marker genes (*Lgr5* for ISC, *Sox9* for Paneth cell, *Ngn3* for enteroendocrine cell, *Hnf1* for enterocyte, and *Klf4* for goblet cell) in untreated organoids and those treated with mecamylamine for seven days. These analyses revealed that the levels of all marker gene transcripts were significantly downregulated compared with the untreated control (Figure 2C). The results indicate that nAChR signaling is involved in organoid growth and differentiation.

### 2.3. Wnt/Frizzled Pathway Activation Is Altered by Nicotine and Mecamylamine Treatment of Organoids

**Figure 3:**
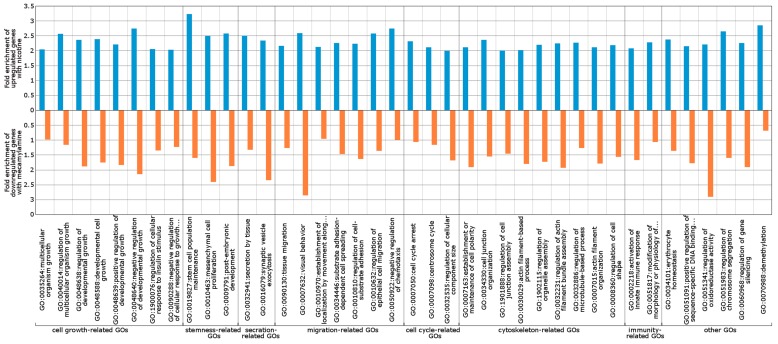
Classification of genes upregulated in response to nicotine and downregulated in response to mecamylamine. Bio-process gene ontology (GO) terms of the differentially expressed genes were mapped by DAVID analysis. Bar graphs show the Fold enrichment score output from DAVID analysis. The blue bar and upper axis indicate fold-enrichment scores for upregulated genes with nicotine and the orange bar and lower axis indicate fold enrichment scores for downregulated genes with mecamylamine.

**Figure 4:**
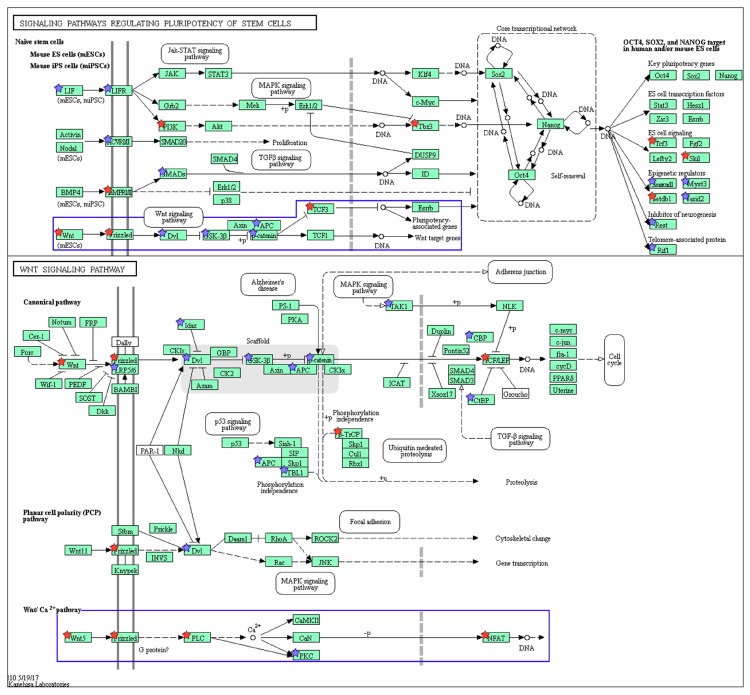
Wnt signaling pathway (mmu04310) maps and Signaling pathways regulating pluripotency of stem cells (mmu04550) derived from DAVID analysis. Green boxes and arrows indicate the genes and interactions in the pathway. +p and −p denote phosphorylation and dephosphorylation, respectively. The genes with red stars are those that were upregulated with nicotine treatment and downregulated with mecamylamine. Additionally, the genes with blue stars represent those that were upregulated in response to nicotine and not downregulated with mecamylamine treatment. The sub-pathways related to the canonical and non-canonical Wnt pathways are highlighted with a blue box.

**Figure 5:**
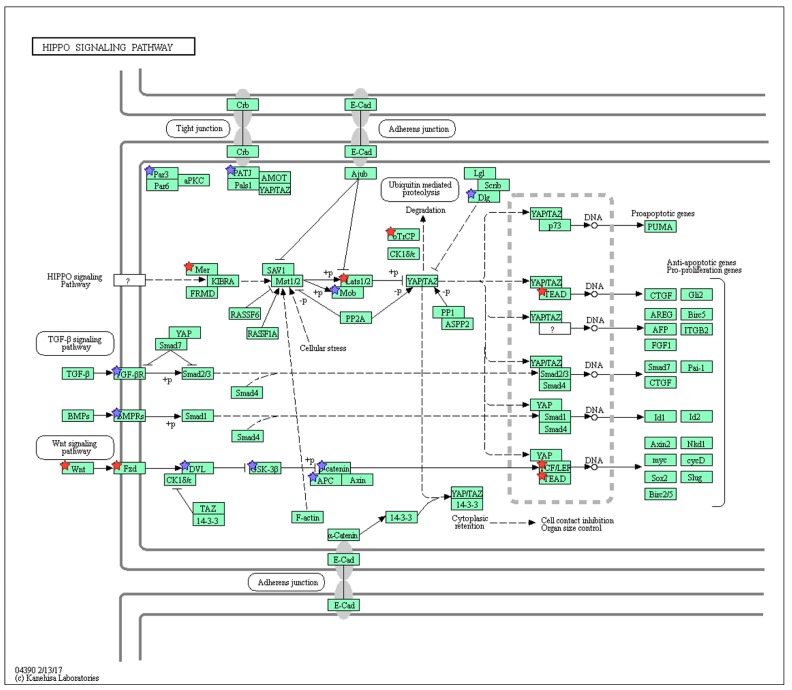
Hippo signaling pathway (mmu04390) maps derived from DAVID analysis. Green boxes and arrows indicate the genes and interactions in the pathway. +p and −p denote phosphorylation and dephosphorylation, respectively. The genes with red stars are those that were upregulated with nicotine treatment and downregulated with mecamylamine. Additionally, the genes with blue stars represent those that were upregulated by nicotine and not downregulated with mecamylamine treatment.

During the past decade, an increasing number of genes potentially involved in nicotine addiction have been identified in both the central and peripheral nervous systems [26]. However, the molecular mechanisms underlying the nicotinic effect on intestinal epithelium remain largely unclear. To address this issue and identify the major underlying biological themes, we carried out RNA sequence (RNA-Seq) analysis (NCBI SRA accession number PRJNA350576). The reads were mapped to the mouse genome mm10 reference assembly and expression fold-changes were calculated with FPKM values. Genes with two-fold differences in expression compared with untreated organoids were defined as differentially expressed. On this basis, 1539 differentially expressed genes were assessed using the automated DAVID systems biology tool [27] and forty gene ontologies (GOs) for Biological Processes and eight KEGG pathways with *p* < 1 × 10^−3^ were identified. The enriched GOs were categorized into cell growth-related, stemness-related, secretion-related, migration related, cell cycle-related, cytoskeleton-related, immunity-related, and others by parent GO terms (Figure 3). The most enriched GO term was “stem cell population maintenance” (GO:0019827) included in the stemness-related GOs (Figure 3), which was concordant with the KEGG pathway analysis output “Signaling pathways regulating pluripotency of stem cells” (mmu04550) as the most enriched pathway (2.88-fold enrichment, Table 1). From the KEGG analysis, “Wnt signal pathway” (mmu04310)-related ligands and receptors were upregulated, and more than 40% of upregulated genes were related to Wnt signaling at both three and seven days after nicotine treatment (Figure 4). The “Thyroid hormone pathway” (mmu04919) was 2.84-fold enriched (Table 1). However, while expression of thyroid hormone and its receptor were not increased, the transcription factors, *Med1*, *Med14*, *Stat1*, *Sin3a*, and *Kat2b* were 11.34-, 4.53-, 3.67-, 3.22-, and 2.55-fold upregulated (Table S1), suggesting that the regulation of expression of these transcription factors was coordinated with other pathways. 50% of genes in the “Hippo signaling pathway” (mmu04390), which was 2.13-fold enriched in upregulated genes (Table 1), overlapped the “Wnt signaling pathway” (mmu04310) (Figure 5). In the “Wnt signaling pathway,” which was 2.06-fold enriched in upregulated genes (Table 1), we found that activation both β-catenin-dependent and -independent pathways was increased by nicotine treatment (Figure 4). Further analysis revealed that *Wnt5a* was highly expressed after treatment with nicotine at day 7 (Table 2). Some putative Frizzled receptors for Wnt5a [28,29,30], *Frizzled-4* and *Frizzled-5*, but not *Frizzled-2*, were 1.99- and 1.80-fold upregulated (Table 2). As shown in Table 2, the amount of *Wnt5a* and *Nuclear factor of activated T cells 1* (*Nfatc1*), which are the starting point and target of β-catenin-independent pathway of Wnt signaling, respectively, were shown to be increased by nicotine treatment at day 7.

We also carried out RNA-Seq analysis after treatment with mecamylamine (NCBI SRA accession number PRJNA350576) (Table S2). For DAVID analysis, genes expressed less than 0.66-fold according to FPKM values were selected as differentially expressed genes and used to calculate the “Fold enrichment” value for the pathways, as described for nicotine treatment. In Figure 4, both downregulated genes with mecamylamine and upregulated genes with nicotine are marked in red. The pathway enrichment analysis for mecamylamine-downregulated genes included “Signaling pathways regulating pluripotency of stem cells” (mmu04550) (Table 3). Wnt ligands were downregulated with mecamylamine in this pathway and the “Wnt signaling pathway” network diagram showed that the non-canonical Wnt pathway was markedly downregulated in response to mecamylamine (Table 2 and Table 3). Based on these results, we focused on the non-canonical Wnt signaling pathway activated by Wnt5a.

**Figure 6:**
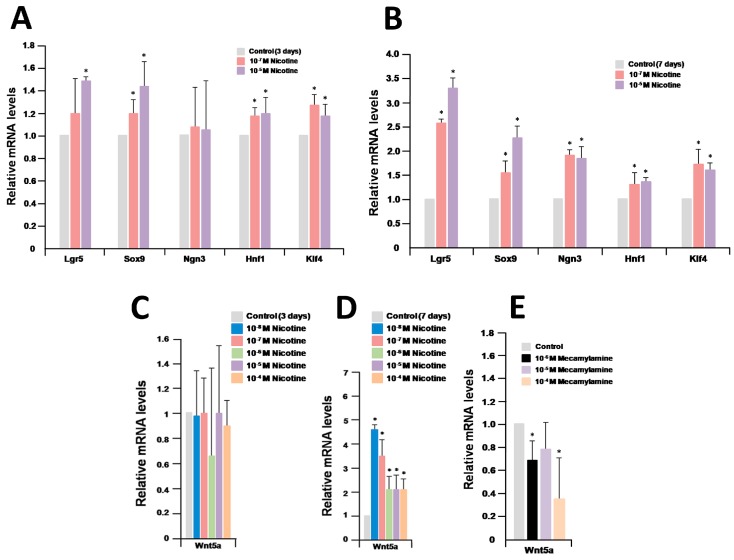
Relative quantification of marker genes and *Wnt5a* after treatment with nicotine and mecamylamine. (**A**,**B**) Relative quantification of marker genes after treatment with nicotine for three days and seven days; (**C**,**D**) relative quantification of *Wnt5a* after treatment with nicotine for three days and seven days; (**E**) relative quantification of *Wnt5a* after treatment with mecamylamine for seven days; (**A**–**E**) the results are based on three independent experiments and are expressed as mean values ± SD. An asterisk indicates a statistically significant difference from untreated control organoids (Student’s *t*-test, * *p* < 0.05).

We carried out RNA-Seq analysis after treatment with nicotine and mecamylamine and found that five marker genes and the *Wnt5a* gene were upregulated after treatment with nicotine, but downregulated after treatment with mecamylamine (Table 2). These data were validated by quantitative RT-PCR analyses. The expression levels of the marker genes were confirmed to be similar between RNA-Seq and quantitative RT-PCR analyses (Figure 2C and Figure 6A,B). Quantitative RT-PCR analysis of organoids treated with nicotine for three days revealed that the expression of *Wnt5a* did not change compared with control organoids (Figure 6C). In contrast, after treatment with nicotine for seven days, the expression of the gene was enhanced in nicotine-treated organoids (Figure 6D). As a result, *Wnt5a* expression was confirmed to be significantly upregulated in nicotine-treated organoids for seven days. Furthermore, *Wnt5a* expression was confirmed to be significantly downregulated in mecamylamine-treated organoids at concentrations of 10^−6^ M and 10^−4^ M for seven days (Figure 6E). In all these analyses, the RNA-Seq and quantitative RT-PCR analyses were strongly correlated, providing additional confidence in the interpretations.

### 2.4. Recombinant Wnt5a Enhances Organoid Growth and Differentiation and Its Effect Is Inhibited By IWP-2

**Figure 7:**
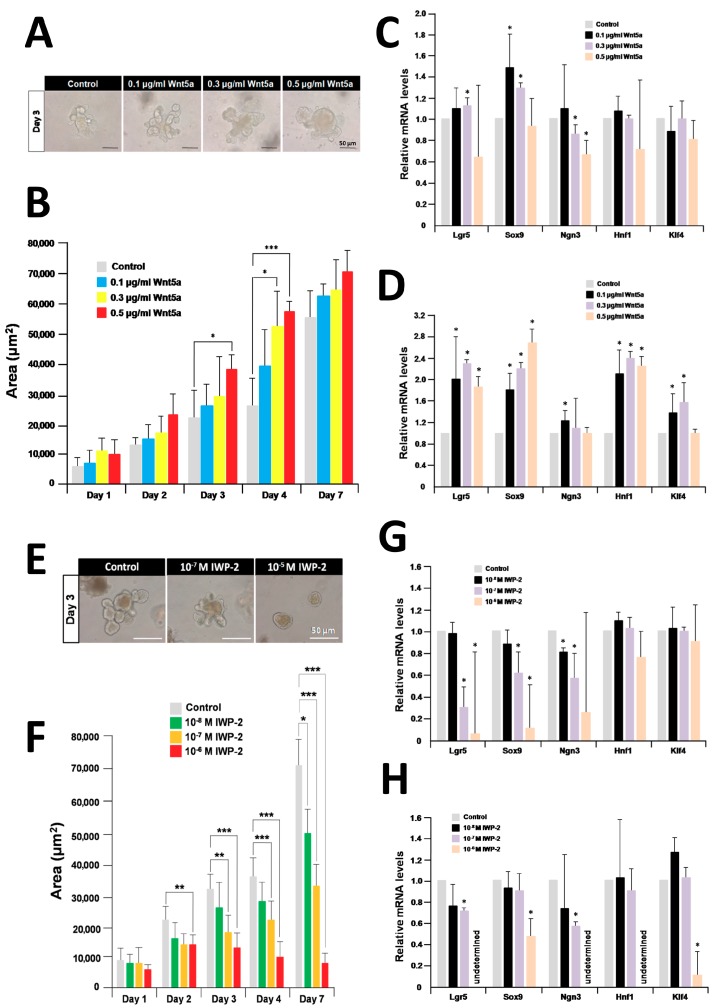
Effects of recombinant Wnt5a and IWP-2 on organoid growth and differentiation. (**A**) Micrographs of organoids after treatment with 0.1 μg/mL, 0.3 μg/mL, and 0.5 μg/mL recombinant Wnt5a for three days of culture; (**B**) effect of recombinant Wnt5a on the size of cultured organoids; (**C**) relative quantification of marker genes after treatment with recombinant Wnt5a for three days; (**D**) relative quantification of marker genes after treatment with recombinant Wnt5a for seven days; (**E**) micrographs of organoids after treatment with 10^−7^ M and 10^−5^ M IWP-2 for three days; (**F**) effect of IWP-2 on the size of cultured organoids; (**G**) relative quantification of marker genes after treatment with IWP-2 for three days; (**H**) relative quantification of marker genes after treatment with IWP-2 for seven days; (**B**,**F**) each sample represents an average of three independent experiments. Error bars represent the SD of the mean. An asterisk indicates a statistically significant difference from untreated control organoids (Mann–Whitney test, * *p* < 0.05, ** *p* < 0.005, and *** *p* < 0.0005). (**C**,**D**,**G,H**) The results are based on three independent experiments and expressed as mean values ± SD. An asterisk indicates a statistically significant difference from untreated control organoids (Student’s *t*-test, * *p* < 0.05).

To evaluate the effect of Wnt5a on the growth and differentiation of organoids, we treated organoids with recombinant Wnt5a at concentrations of 0.1 μg/mL, 0.3 μg/mL, and 0.5 μg/mL over a seven-day period [31]. Recombinant Wnt5a slightly enhanced organoid growth over control organoids (Figure 7A). The enhancement of organoid growth occurred in a dose-dependent manner until day 7 (Figure 7B). Organoids treated with 0.5 μg/mL recombinant Wnt5a for three days, and with 0.3 μg/mL and 0.5 μg/mL recombinant Wnt5a for four days were confirmed to be significantly enhanced in growth compared with control ones (Figure 7B). Quantitative RT-PCR analysis of organoids treated with recombinant Wnt5a for three days revealed that the levels of *Lgr5* and *Sox9* transcripts were slightly enhanced compared with the untreated control (Figure 7C). In contrast, the levels of *Lgr5*, *Sox9*, and *Hnf1* transcripts were approximately doubled in organoids treated with Wnt5a for seven days compared with the untreated control (Figure 7D).

We treated organoids with IWP-2, which is an inhibitor of O-acylation, a posttranslational modification that is required for all Wnt secretion [32]. Organoid growth was dramatically inhibited by 10^−7^ M and 10^−5^ M IWP-2 (Figure 7E). The growth of organoids treated with 10^−6^ M IWP-2 for two days was inhibited in a dose-dependent manner compared with controls (Figure 7F). Next, we compared the expression levels of selected marker genes in IWP-2-treated and untreated organoids for three or seven days using quantitative RT-PCR. *Lgr5*, *Sox9*, and *Ngn3* transcripts were significantly downregulated in organoids treated for three days with 10^−6^ M IWP-2 compared with untreated organoids (Figure 7G). Interestingly, the expression of all marker genes was dramatically inhibited by treatment with 10^−6^ M IWP-2 for seven days (Figure 7H).

### 2.5. Recombinant Wnt5a Rescues the Organoids Treated with Mecamylamine

**Figure 8:**
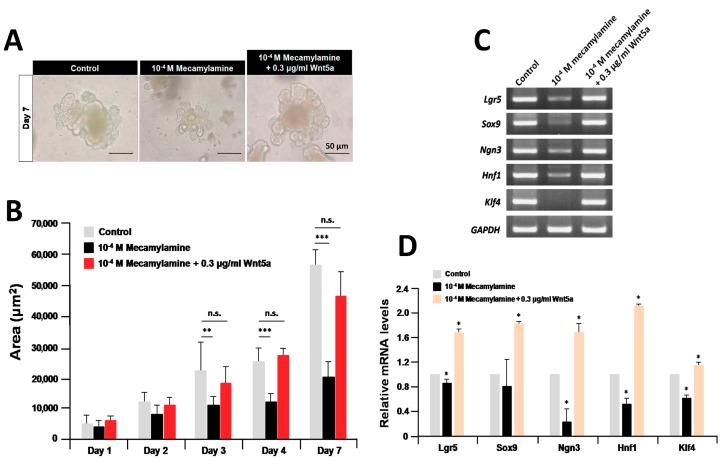
Rescue of organoid growth and differentiation after co-treatment with mecamylamine and recombinant Wnt5a. (**A**) Micrographs of organoids after treatment with 10^−4^ M mecamylamine and 10^−4^ M mecamylamine/Wnt5a for seven days; (**B**) effect of 10^−4^ M mecamylamine and 10^−4^ M mecamylamine/Wnt5a on the size of cultured organoids. Each sample represents an average of three independent experiments. Error bars represent the SD of the mean. An asterisk indicates a statistically significant difference from untreated control organoids (Mann–Whitney test, n.s., not significant, ** *p* < 0.005, and *** *p* < 0.0005). (**C**) Effect of 10^−4^ M mecamylamine and 10^−4^ M mecamylamine/Wnt5a on the expression of ISC markers and epithelium in a seven-day culture of organoids; (**D**) relative quantification of marker genes after treatment with 10^−4^ M mecamylamine and 10^−4^ M mecamylamine/Wnt5a for seven days. The results are based on three independent experiments and are expressed as mean values ± SD. An asterisk indicates a statistically significant difference from untreated control organoids (Student’s *t*-test, * *p* < 0.05).

The findings that nicotine enhanced the expression of *Wnt5a*, and that recombinant Wnt5a enhanced organoid growth and differentiation, suggest coordination with nAChR signaling. If the function of Wnt5a in organoid growth and differentiation depends on nAChR signaling activity, it might be possible to rescue organoid growth and differentiation by exogenous application of recombinant Wnt5a while simultaneously co-treating with mecamylamine. To examine this possibility, organoids were co-treated with 0.3 μg/mL recombinant Wnt5a and 10^−4^ M mecamylamine. Organoid growth was dramatically inhibited by 10^−4^ M mecamylamine compared with the untreated control, as expected (Figure 8A). However, treatment with both drugs simultaneously resulted in organoid growth at day 7 that was similar to controls (Figure 8A). Time-course analysis revealed that the inhibition of organoid growth following treatment with 10^−4^ M mecamylamine was completely rescued by co-treatment with 0.3 μg/mL recombinant Wnt5a (Figure 8B). Furthermore, RT-PCR analysis revealed that the expression levels of all marker gene transcripts after co-treatment with both drugs for seven days reached the levels of controls (Figure 8C). We confirmed that the expression of all marker gene transcripts after co-treatment with both drugs for seven days surpassed above the control by using quantitative RT-PCR (Figure 8D). Collectively, the results indicated that recombinant Wnt5a rescued the growth of mecamylamine-treated organoids.

### 2.6. α2/β4 nAChR Is Localized in Paneth Cells

**Figure 9:**
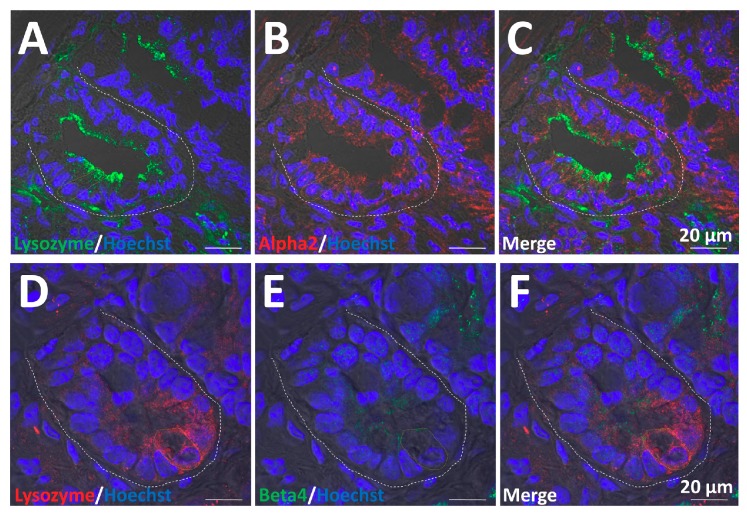

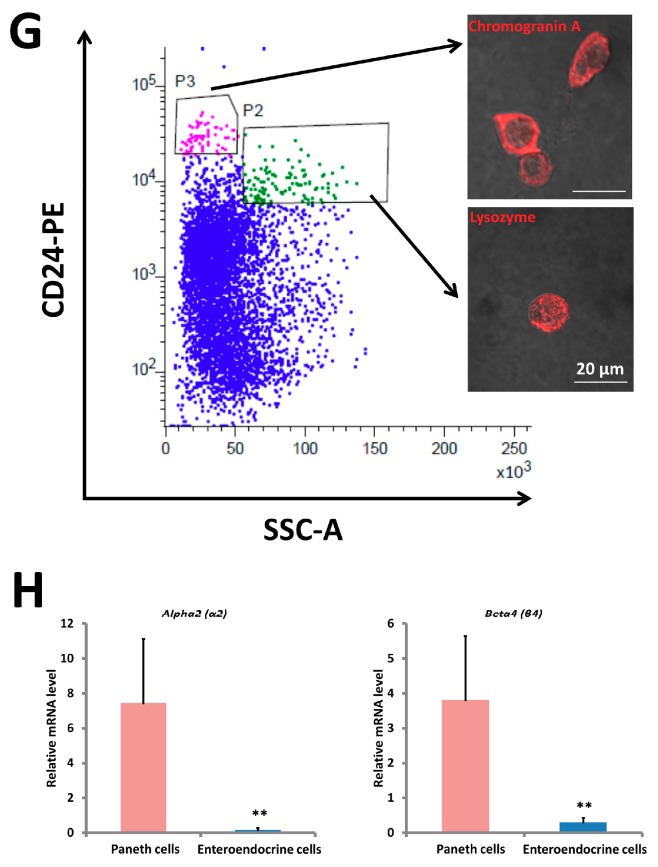
Co-localization and gene expression of α2 and β4 in Paneth cells. (**A**) Visualization of lysozyme (green) in crypts; (**B**) visualization of α2 (red) in crypts; (**C**) merged visualization of (**A**) and (**B**); (**D**) visualization of lysozyme (red) in crypts; (**E**) visualization of β4 (green) in crypts; (**F**) merged visualization of (**D**) and (**E**). White dotted lines indicate the crypt region. Orange dotted lines indicate the typical cell. In all panels, nuclei were stained with Hoechst 33342 (blue). Bars in (**A**–**F**) represent 20 μm. (**G**) FACS plot of dissociated single cells from organoid tissues. Two CD24 bright populations differ by side-scatter (SSC) pattern. Sorted CD24 ^high^/SSC ^low^ (P3) and CD24 ^high^/SSC ^high^ (P2) cells are subsequently stained. CD24 ^high^/SSC ^low^ cells are positive for the enteroendocrine marker chromogranine A (top right), whereas CD24 ^high^/SSC ^high^ cells are positive for the Paneth marker lysozyme (bottom right). (**H**) CD24 ^high^/SSC ^high^ (P2) and CD24 ^high^/SSC ^low^ (P3) cells were examined for transcripts of *α2* (*Alpha2*) and *β4* (*Beta4*) with quantitative RT-PCR. The results are based on three independent experiments as mean values ± SD. The statistical significance was calculated with Student’s *t*-test (** *p* < 0.005).

Paneth cells have a stem cell niche function [33]. To determine if the α2/β4 nAChR subtype is localized in Paneth cells, we carried out double immunohistochemical staining using antibodies against α2 or β4, and the Paneth cell marker protein, lysozyme. As shown in Figure 9A–F, the α2 and β4 subunits co-localized with lysozyme. The results indicate that the α2/β4 nAChR subtype is localized in Paneth cells. In addition, we performed to isolate Paneth cells with a cell sorter using the mouse anti-CD24-PE antibody [33]. We obtained two types of cells, CD24 ^high^/SSC ^high^ (P2) and CD24 ^high^/SSC ^low^ (P3) cells, using fluorescence-activated cell sorting (FACS) (Figure 9G). Then, we examined immunostaining analysis to confirm each cell type. CD24 ^high^/SSC ^low^ cells (P3) are positive for the enteroendocrine marker chromogranine A (Figure 9G, top right), whereas CD24 ^high^/SSC ^high^ cells (P2) are positive for the Paneth marker lysozyme (Figure 9G, bottom right). We conclude that CD24 ^high^/SSC ^low^ cells (P3) are enteroendocrine cells and CD24 ^high^/SSC ^high^ cells (P2) are Paneth cells, respectively. In agreement with the immunostaining analysis, the transcripts of the *α2* (*Alpha2*) and *β4* (*Beta4*) were exclusively confirmed to be expressed in Paneth cells. The results also indicate that the genes encoding α2 (Alpha2) and β4 (Beta4) are selectively expressed in Paneth cells of organoids.

## 3. Discussion

ACh and its receptors are among the best characterized neurotransmitter/receptor systems [34,35,36,37]. There are two principal forms of AChRs: mAChRs and nAChRs. Besides nerve cells, it is now well established that ACh is a ubiquitous molecule that plays important roles in various aspects of cell biology and homeostasis in keratinocytes [38], epithelial cells [21,39,40], and endothelial cells [41]. However, the physiological role of nAChRs in intestinal epithelial cells remains poorly understood.

In mice, intestinal epithelial cells are newly generated in crypts and are lost following apoptosis at the tip of the villi within five days. Self-renewing Lgr5-positive ISCs reside near the crypt base [2]. In 2009, the first culture system for an intestinal epithelial organoid (mini-gut) was established using Lgr5-positive stem cells [4]. Furthermore, a single ISC forms 3D crypt structures [4]. These mini-guts comprise all types of differentiated cells normally present in the gut, but not nerve, immune, and mesenchymal cells [4]. Importantly, single murine ISCs can be expanded in culture over extended periods as genetically and phenotypically stable epithelial organoids [4,21].

In this study, we showed that the nicotinic receptor agonist, nicotine, slightly increased organoid size and marker gene expression. The analysis of the pharmacological effects of nicotine revealed that endogenous ACh enhances proliferation and differentiation via nAChRs, probably facilitating interactions between nAChR signaling and other regulatory networks. We also examined the effect of mecamylamine on the growth and differentiation of organoids, and found that this anti-nicotinic agent had an antagonistic effect compared with nicotine. These observations implicate ACh receptors in the control of stem cell proliferation and differentiation in the gut.

Our RNA-Seq experiments using nicotine and mecamylamine simulations showed that differential expression of *Wnt5a* is one of the key events downstream of nAChR signaling. However, the endogenous expression level of *Wnt5a* is weak. Instead, we showed that recombinant Wnt5a increases organoid growth and activates the expression of marker genes over three to seven days of treatment. We also examined the effect of IWP-2 on the growth and differentiation of organoids, and found that this anti-Wnt agent had an inhibitory effect compared with recombinant Wnt5a. These findings are consistent with a role of Wnt5a in organoid growth and differentiation, and the effect of nicotine on organoid growth and differentiation appears to be linked to Wnt5a activity. Wnt5a activates β-catenin-independent pathways such as the planar cell polarity (PCP) and Ca^2+^ cascades [42,43]. The PCP pathway was originally identified in *Drosophila*, where it is involved in the regulation of tissue polarity, cell migration, and cytoskeleton arrangement [42]. Although we found that the PCP pathway was activated after treatment with nicotine, downstream signaling outputs were not clearly observed. Thus, we concluded that the PCP pathway is not coordinated with nAChR signaling. The Ca^2+^ pathway triggered by specific Wnt proteins such as Wnt5a leads to an increase in intracellular Ca^2+^ and activation of calcium-sensitive enzymes, such as calcineurin and Ca^2+^/calmodulin-regulated kinase II [44,45]. Although it is commonly believed that Wnt5a can induce a Ca^2+^ signal [43], the Wnt/Ca^2+^ pathway in itself is not generally considered to be a regulator of stem cell proliferation and differentiation [46]. Stimulation of the calcium-sensitive enzymes leads to the activation of *Nfatc* transcription factor protein in many cell types [47,48]. Our RNA-Seq data indicated that nicotine can also activate *Nfact1* via a non-canonical pathway through activation of Wnt5a in organoids. It is probable that the Wnt/Ca^2+^ pathway activated by Wnt5a linked to nAChR signaling may control stem cell function in Lgr5-positive ISCs.

Our RNA-Seq data also suggest that activation of the β-catenin-dependent pathway is altered by nicotine treatment. One of the canonical ligands, Wnt9b, but not Wnt3, was upregulated in the RNA-Seq analysis. Epithelial Wnt9b is known to be involved in the maintenance of the ISC niche [33]. Another stem cell regulating network, the Hippo pathway, was upregulated after treatment with nicotine. In ISCs, the Hippo pathway inhibits YAP activity by phosphorylation leading to the subsequent cytosolic retention of YAP [49], which directly binds to β-catenin and inhibits canonical Wnt signaling [49]. It is probable that the slightly increased organoid growth in response to nicotine treatment results from activation of the canonical Wnt and Hippo pathways. Cao and co-workers revealed broad effects on gestational nicotine treatment on the cell adhesion system that modified genes in the neurexin, immunoglobulin, cadherin, and adhesion G-protein coupled receptor superfamilies in limbic brain regions [26]. Probably, non-neuronal cholinergic systems are functionally expressed independently of cholinergic innervation and modify or even control phenotypic cell functions in the mouse intestine.

To obtain further insights into the role of nAChRs in organoids, we examined whether recombinant Wnt5a can rescue the effect of mecamylamine. Treatment with both drugs simultaneously resulted in organoid growth and differentiation similar to controls, indicating that nAChR and Wnt5a signaling are coordinated. The exact mechanisms underlying the coordination between nAChR and Wnt signaling in proliferation and differentiation of Lgr5-positive ISCs are still unknown, but our rescue experiment suggests that nAChR signaling is indispensable for ISC activity in response to increased Wnt signaling.

Although it is generally believed that Wnt5a is localized in the mesenchymal layer in adult gut [14], the localization and function of Wnt5a in gut epithelium is controversial. Bakker and co-workers [50] showed that the adult mouse intestine is not affected by induced Wnt5a expression, and histological aberrations or changes in β-catenin signaling were not detected. In other contexts, Wnt5a can potentially interact with multiple signaling pathways to modulate cell proliferation, migration, and differentiation [51,52,53,54]. Supportively, levels of endogenous Wnt5a expression in adult intestine are relatively low compared to levels in developing gut [14,19,55,56]. Our RNA-Seq data demonstrated that *Wnt5a* expression is extremely low in organoids. Wnt5a has been shown to be often upregulated typically under stress, such as wounding and tumorigenesis in the intestine [20,57,58]. It seems that epithelial Wnt5a signaling is not critical for proliferation and differentiation of Lgr5-positive ISCs under normal conditions in adults, as long as *Wnt5a* is expressed in the intestinal mesenchyme rather than epithelium. As our results indicate that recombinant Wnt5a increases the expression of the *Lgr5* ISC marker and epithelial cell markers (*Sox9* for Paneth cells and *Hnf1* for enterocytes), we conclude that exogenous Wnt5a enhances organoid size slightly after three to four days and *Lgr5* expression after seven days, without overtly affecting Lgr5-positive ISC function. Considering this, we speculate that there is a threshold level of endogenous ACh above which more profound effects follow Wnt5a induction. Furthermore, as increased *Wnt5a* expression is typically observed after treatment with nicotine, endogenous ACh may be an additional player involved in the postnatal activities of Wnt5a.

Various combinations of nAChR subunits exhibit a wide range of physiological and pharmacological profiles, and are differentially expressed throughout the neuronal and non-neuronal systems [7,59]. Our experiments with antibodies against nAChR subunits is the first description of the α2/β4 subtype in the crypt region, but not villi, implying potentially novel functions, such as regulation of stem cell proliferation and differentiation. Importantly, we observed overlapping fluorescence of α2- and β4-specific antibodies with an anti-lysozyme antibody that recognizes Paneth cells. Paneth cells are a critical component of the ISC niche both in vivo and in vitro [2,33]. Flow cytometric analysis and cell sorting using the mouse CD24 antibody indicated that Paneth cells exclusively exhibited the expression of the genes encoding α2 and β4. These findings suggest that the function of Lgr5-positive ISCs is, at least in part, under the control of an independent epithelial cholinergic system linked to the α2/β4 nAChR subtype.

**Figure 10:**
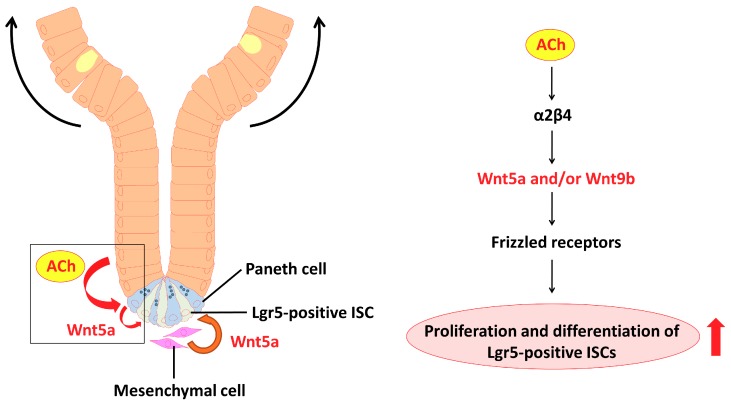
Model depicting the proposed role of nAChR signaling in intestinal stem cell function. Non-neuronal ACh activates the nicotinic receptor α2/β4 localized in the Paneth cells to modulate the expression levels of *Wnt5a* and/or *Wnt9b*. The Wnts then mediate Wnt pathway activity through various frizzled receptors. Eventually, proliferation and differentiation of stem cells are enhanced.

Our data suggest in part that the regulation of ISCs in normal adult mouse crypts is linked to upregulation of nAChR-driven *Wnt* expression. According to the model (Figure 10), endogenous ACh binds to the α2/β4 nAChR subtype in Paneth cells, and then induces *Wnt5a* and/or *Wnt9b* expression. The Wnts bind to various Frizzled receptors to activate Wnt signaling, and eventually proliferation and differentiation of Lgr5-positive ISCs are enhanced.

In conclusion, our data demonstrate that non-neuronal ACh is involved in the proliferation and differentiation of Lgr5-positive ISCs through a novel combinatorial pathway that regulates the homeostasis of intestinal epithelial cell density. The combinatorial pathway described here may be a common theme in stem cell biology. We also revealed that endogenous ACh released from intestinal epithelium maintains homeostasis of intestinal epithelial cell growth and differentiation via mAChRs [21]. Simultaneous stimulation of both ACh receptor classes may be required to synchronize and balance ionic and metabolic events in a single cell, and crosstalk between these receptors may fine tune the signals emanating from epithelial cells. The characterization of these pathways may clarify the mechanisms underlying developmental processes in the typical crypt–villus unit.

Newly developed medical treatments for human disease usually have limitations such as individual differences among patients, difficulties with prediction of outcomes, and time-consuming drug testing [60,61,62,63]. Organoid cultures based on a specific disease and even on a specific individual are expected to develop into powerful tools for precision therapy [60,64,65]. For example, each component of the non-neuronal cholinergic system can be affected as a key pathogenetic event or secondary event to the disease state. In particular, the fine-tuning of cellular effects mediated by the different subtypes of nAChRs and mAChRs may become impaired. The scientific community has just started to investigate the non-neuronal cholinergic system [21]. A systematic analysis of all components of the non-neuronal cholinergic system in different diseases is practically non-existent and should be performed as soon as possible. This will increase our understanding of the cholinergic properties of non-neuronal cells and in turn lead to optimization of drug therapy.

## 4. Materials and Methods

### 4.1. Mice

This study was approved by the Suntory animal ethics committee (APRV000246, 19 May 2016), and all animals were maintained in accordance with committee guidelines for the care and use of laboratory animals. C57/BL6 mice used for small intestinal crypt isolation were 8–12 weeks of age. Mice were euthanized with CO_2_ asphyxiation.

### 4.2. Crypt Isolation and Crypt–Villus Organoid Culture

Crypt isolation and crypt–villus organoid culture were performed according to the method of Takahashi et al. [21]. Briefly, isolated small intestine was opened longitudinally and washed with cold phosphate-buffered saline (PBS) including penicillin (10,000 units/mL), streptomycin (10,000 μg/mL), and gentamicin sulfate solution (10 mg/mL) (PBS-ABx). The tissue was cut into 5 × 5-mm pieces that were further washed with cold PBS-ABx. The washed tissue fragments were incubated in PBS-ABx containing 2 mM EDTA for 30 min on ice. After removal of the EDTA solution, the tissue fragments were vigorously shaken in cold PBS-ABx. The resultant suspension was passed through a 70-μm cell strainer (BD Biosciences, Bedford, MA, USA) to remove residual villous material, and the isolated crypts were then centrifuged (390× *g*, 3 min, 4 °C). The final fraction consisted of essentially pure crypts, and was used for culture. Isolated crypts were counted and pelleted by centrifugation at 290× *g* for 3 min at 4 °C. A total of 100 crypts were mixed with 40 μL of Matrigel (BD Biosciences) and plated in 24-well plates pre-warmed to 37 °C. After Matrigel polymerization, 500 μL of crypt culture medium containing growth factors (20 ng/mL mouse EGF (R&D Systems, Minneapolis, MN, USA), 500 ng/mL R-spondin (R&D Systems), and 100 ng/mL Noggin (R&D Systems) was added. The organoids were maintained at 37 °C in a humidified atmosphere with 5% carbon dioxide. The culture medium was changed every other day.

### 4.3. Subculture of Crypt–Villus Organoids

Old culture medium was removed and the Matrigel was washed with cold PBS three times. After adding 1 mL BD Cell Recovery Solution (BD Biosciences), the Matrigel was mechanically peeled off and the remaining solution was incubated for 15 min on ice with shaking. Isolated organoids were pelleted by centrifugation at 195× *g* for 1 min at 4 °C. The pellet was mechanically dissociated into single-crypt domains for passaging, and transferred to fresh Matrigel. Passaging was performed every 1–2 weeks with a 1:3 split ratio.

### 4.4. Pharmacological Assay

Crypt–villus organoids contain a non-neuronal cholinergic system [21]. A pharmacological assay was performed with mechanically dissociated single-crypt domains, which were grown as organoids in the presence or absence of nicotine (Nacalai Tesque, Kyoto, Japan), mecamylamine (Tocris Bioscience, St. Louis, MO, USA), recombinant Wnt5a (R&D Systems), and IWP-2 (Santa Cruz Biotechnology, Dallas, TX, USA). We dissolved nicotine, mecamylamine, and recombinant Wnt5a in PBS and dissolved IWP-2 in DMSO (Nacalai Tesque), respectively. For control organoids, regular medium with PBS was used to test the effect of nicotine, mecamylamine, and recombinant Wnt5a. In contrast, regular medium with DMSO was used for control organoids to test the effect of IWP-2. The drugs were administrated directly when seeding single-crypt domains at time-point zero, and the organoids were collected for analysis three or seven days later. The culture medium with or without drugs was replaced every other day. To assay growth, organoid surface area was analyzed with the Image J program (NIH, Bethesda, MD, USA). The effects of the drugs on marker gene expression in Lgr5-ISCs and epithelium (*Sox9*, *Ngn3*, *Hnf1*, and *Klf4*; differentiation markers for Paneth cells, enteroendocrine cells, enterocytes, and goblet cells, respectively) were analyzed by RT-PCR in treated and untreated organoids. The primer sequences used for PCR amplification are shown in Table S3.

### 4.5. RT-PCR

Total RNA from organoid tissues was extracted with TRIzol reagent (Gibco, Penrose, OK, USA) according to the manufacturer’s instructions. The extracted RNA was treated with DNase I (Ambion, Austin, TX, USA) to eliminate genomic DNA from RNA preparations. Total RNA (3 μg) was used as a template for cDNA synthesis. Reverse transcription was performed with SuperScript II and an oligo-dT primer according to the protocol recommended by the manufacturer (Invitrogen, Camarillo, CA, USA). cDNAs encoding nAChR subunits, *Lgr5*, *Sox9*, *Ngn3*, *Hnf1*, *Klf4* were amplified by PCR with designed PCR primers (Table S3). The RT-PCRs were carried out using a GeneAmp PCR System 9700 (Applied Biosystems, Foster City, CA, USA) with conditions as follows: 3 min initial denaturation at 94 °C; followed by 35 cycles of 94 °C for 30 s, 55 °C for 30 s, and 72 °C for 1 min, and then one final extension step of 72 °C for 5 min. The expression of glyceraldehyde 3-phosphate dehydrogenase (*GAPDH*) was used as an internal control.

### 4.6. Quantitative RT-PCR Analysis

Organoids were treated with nicotine, mecamylamine, Wnt5a, and IWP-2 in a culture medium for three or seven days at 37 °C. Quantitative RT-PCR for *Lgr5*, *Sox9*, *Ngn3*, *Hnf1*, *Klf4* was undertaken using SYBR Green master mixture (Bio-Rad, Hercules, CA, USA), according to the manufacturer’s procedure in triplicate. The quantitative RT-PCR was carried out on a CFX96^TM^ Real-Time System (Bio-Rad) with conditions as follows: 30 s polymerase activation and DNA denaturation, followed by 45 cycles of 95 °C for 10 s and 55 °C for 30 s; then 65 °C for 5 s, and then 95 °C for 50 s for melt-curve analysis. *GAPDH* was amplified as an internal control. All primers for quantitative RT-PCR are presented in Table S4. For relative quantification of gene expression, the comparative C_T_ method was used.

### 4.7. Immunohistochemistry

Gut tissue sample was embedded in Tissue-Tek OCT compound (Qiagen, Tokyo, Japan), frozen at −20 °C, and then cut serially into 10-μm-thick sections with a cryostat (Thermo Scientific, Waltham, MA, USA). For immunohistochemical analysis, the sections were fixed with 4% paraformaldehyde (PFA) (Nacalai Tesque) for 10 min at room temperature. After three washes with PBS, the sections were treated with 0.1% sodium borohydride (Nacalai Tesque) for 10 min at room temperature to reduce autofluorescence, and were again washed three times with PBS. The sections were then treated with 0.1% Triton X-100 (Nacalai Tesque) for 10 min at room temperature, and washed with PBS three times. The sections were incubated with blocking solution containing 1% BSA (Sigma, Foster City, CA, USA) for 10 min at room temperature followed by incubation for 60 min at room temperature in primary antibodies diluted appropriately in blocking solution. The primary antibodies used for immunohistochemistry were as follows: anti-nicotinic ACh receptor α2 (1:1000; Alomone Labs, Jerusalem, Israel), anti-nicotinic ACh receptor β4 (1:1000; Abcam, Tokyo, Japan), two types of anti-lysozyme antibody (1:1000; Abcam, Cat No. ab36362 and ab108508), and anti-chromogranin A antibody (1:1000; Abcam). After incubation, the sections were washed three times with PBS and further incubated with secondary antibodies diluted with blocking solution. The secondary antibodies used were Alexa Fluor 488 goat anti-rabbit IgG (1:1000), Alexa Fluor 546 goat anti-rabbit IgG (1:1000), Alexa Fluor 488 donkey anti-goat IgG (1:1000), Alexa Fluor 568 donkey anti-goat IgG (1:1000), Alexa Fluor 488 goat anti-mouse IgG (1:1000), and Alexa Fluor 568 goat anti-mouse IgG (1:1000) (all from Molecular Probes, Camarillo, CA, USA). Nuclei were stained with Hoechst 33342 (1:1000; AnaSpec, Fremont, CA, USA). Control sections were labeled in the absence of primary antibodies. Sections were mounted in Mowiol mounting medium (Mowiol 4-88; Sigma) under a cover glass, and observed by confocal immunofluorescence microscopy (FV 1000; Olympus, Tokyo, Japan).

Single-cell immunohistochemical staining was also conducted as described previously [33]. Briefly, single cells were isolated from organoids using FACS analysis, fixed in 4% PFA for 10 min at room temperature, and then washed three times for 5 min each in PBS. Next, the cells were incubated in 0.1% TX-100 solution for 10 min, and then washed three times for 5 min each in PBS. The samples were blocked with 1 mL blocking solution for 10 min, and then the primary antibody (anti-lysozyme or chromogranin A antibody) diluted with blocking solution (1:1000) was added followed by incubation overnight at 4 °C. After washing three times for 5 min each in PBS, the secondary antibody (Alexa Fluor 568 goat anti-mouse IgG) was added and the samples were incubated for 30 min at room temperature. After a final wash in PBS, the samples were mounted with Mowiol and observed by confocal microscopy.

### 4.8. RNA Sequencing

Total RNA from organoids was extracted from log phase cultures using a RNeasy Mini Kit (Qiagen). The quality of the RNA samples was evaluated using a BioAnalyzer (Agilent Technologies, Foster City, CA, USA) with the RNA6000 Nano Chip. Total RNA (2 μg) from each sample was used to construct cDNA libraries using TruSeq Stranded mRNA (Illumina, Foster City, CA, USA) according to the manufacturer’s instructions. The cDNA library was validated using the BioAnalyzer with DNA1000 Chip and quantified using the Cycleave PCR Quantification Kit (Takara Bio, Shiga, Japan). Single end sequencing using 101 cycles was performed using HiSeq1500 (Illumina) in the rapid output mode. Total reads were extracted with CASAVA v1.8.2 (Illumina). PCR duplications, adaptor sequences, and low-quality reads were removed from the extracted reads as follows: If the first 10 bases of the two reads were identical and the entire reads showed >90% similarity, the reads were considered to be PCR duplicates. Base calling from the 5′ to the 3′ end was stopped when the frequency of accurately called bases dropped to 0.5. The NextGen sequencing data were deposited in the Sequence Read Archive (SRA) database (accession number PRJNA350576). The remaining reads were aligned to mouse reference transcripts (build mm10) from the University of California Santa Cruz (UCSC) genome browser database. The alignment parameters were set to allow a single mutation in alignment and ignore the mismatch penalty for low-quality nucleotides with lower quality than 20. The alignment was performed against the mouse reference genome using Cufflinks (v2.0.10). The reference genome and annotations were downloaded in fasta and gene transfer format (GTF) from the UCSC genome browser database. All the parameters for Cufflinks were set to default value. Differentially expressed genes were selected if they showed a greater than two-fold change between the two groups and were subsequently assessed by DAVID [27]. From DAVID analysis, differentially expressed genes were categorized into Gene Ontology (GO) and KEGG (Kyoto Encyclopedia of Genes and Genomes) pathways. Enriched GOs and pathways were selected from third-level Biological Process (BP-3) and KEGG categories that had more than 10 upregulated and downregulated genes (1 × 10^−3^), and annotated as upregulated and downregulated pathways in response to nicotine and mecamylamine treatment, respectively. For further analysis, the ‘Count’ and ‘Fold enrichment’ values calculated with DAVID were utilized.

### 4.9. Flow Cytometry Analysis and Cell Sorting

FACS analysis and cell sorting were performed as described previously [33]. In brief, organoids were dissociated with TrypLE express (Gibco) for 10 min at 37 °C at room temperature. Dissociated cells were passed through 20-μm cell strainer (Sysmex Partec GmbH, Goerlitz, Germany) and washed with PBS. Cells were stained with PE-conjugated anti-CD24 antibody (BD Biosciences) for 30 min at 4 °C in dark, and analyzed by FACSMelody (BD Biosciences). Viable epithelial single cells or doublets were gated by forward scatter, side scatter and pulse-width parameter, and negative staining for 7-AAD (BD Biosciences).

### 4.10. Statistical Analysis

Comparisons between two groups of data were made with the Mann–Whitney test or Student’s *t*-test, as applicable. The data were compared between the treated organoids and the non-treated controls in organoid growth by Mann–Whitney test for non-normal variables and unpaired *t*-test for normally distributed variables in quantitative RT-PCR. Data and statistical analyses were performed with Microsoft Excel. Data are presented as means ± standard deviation (means ± SD). All experiments were repeated at least three times.

## Figures and Tables

**Table 1:** Upregulated pathways after nicotine treatment.

KEGG Pathway ID	KEGG Pathway Designation	Number of Genes	Fold Enrichment
mmu04550	Signaling pathways regulating pluripotency of stem cells	26	2.88
mmu04919	Thyroid hormone signaling pathway	21	2.84
mmu04390	Hippo signaling pathway	21	2.13
mmu04310	Wnt signaling pathway	19	2.06
mmu04010	MAPK signaling pathway	27	1.63
mmu04144	Endocytosis	29	1.59
mmu04015	Rap1 signaling pathway	25	1.79
mmu04810	Regulation of actin cytoskeleton	22	1.57

**Table 2:** Differential expression patterns of marker genes and genes expressed in the Wnt pathway and other stem cell pathways, after treatment with nicotine and mecamylamine.

Accession	Gene Name	Nicotine (Fold Change *)				Mecamylamine (Fold Change *)
		10^−7^ M(3 Day)	10^−5^ M(3 Day)	10^−7^ M(7 Day)	10^−5^ M(7 Day)	10^−6^ M (7 Days)
NM_019827	*Gsk3β*	1.09	1.05	1.64	1.71	1.00
NM_007614	*Ctnnb1*	0.91	0.78	1.61	1.88	0.84
NM_007561	*Bmpr2*	1.01	1.06	1.60	1.70	0.98
NM_007889	*Dvl3*	1.08	1.02	1.74	1.73	1.10
NM_001005784	*Patj*	0.39	0.62	3.74	3.88	1.06
NM_026735	*Mob1b*	1.15	1.10	2.27	2.37	0.83
NM_007862	*Dlg1*	1.19	1.18	1.74	1.96	1.66
NM_001109752	*Dlg4*	16.37	7.20	2.28	2.62	0.80
NM_145571	*Mob1a*	1.04	1.05	1.64	1.84	0.96
NM_001166585	*Tead1*	1.65	1.30	1.95	1.64	1.13
NM_010690	*Lats1*	1.10	1.08	1.64	1.62	0.98
NM_001164112	*Nfatc1*	1.20	0.87	1.60	1.99	0.00
NM_009521	*Wnt3*	1.10	1.13	1.10	1.03	1.05
NM_001256224	*Wnt5a*	1.30	1.37	4624.5	3885.7	0.00
NM_011719	*Wnt9b*	1.30	1.25	1.84	1.41	0.29
NM_020510	*Frizzled-2*	1.16	1.14	1.11	0.95	0.89
NM_008055	*Frizzled-4*	0.98	0.94	1.82	1.99	0.83
NM_022721	*Frizzled-5*	1.05	0.89	1.84	1.80	0.97
NM_001162494	*Frizzled-6*	1.02	1.08	2.01	1.88	1.02
NM_010195	*Lgr5*	1.16	1.09	1.46	1.78	0.53
NM_011448	*Sox9*	1.07	1.33	1.25	1.48	0.27
NM_009719	*Ngn3*	1.30	1.59	0.97	1.39	0.28
NM_009327	*Hnf1*	1.10	1.17	1.19	1.31	0.15
NM_010637	*Klf4*	1.03	1.04	1.31	1.44	0.17

* Fold change is defined as the ratio of the signal of non-treated organoids to that of treated organoids.

**Table 3:** Downregulated pathways after mecamylamine treatment.

KEGG Pathway ID	KEGG Pathway Designation	Number of Genes	Fold Enrichment
mmu04550	Signaling pathways regulating pluripotency of stem cells	16	2.20
mmu04919	Thyroid hormone signaling pathway	9	1.50
mmu04390	Hippo signaling pathway	14	1.70
mmu04310	Wnt signaling pathway	13	1.70
mmu04010	MAPK signaling pathway	25	1.80
mmu04144	Endocytosis	27	1.80
mmu04015	Rap1 signaling pathway	20	1.70
mmu04810	Regulation of actin cytoskeleton	20	1.70

## Supplementary Materials

Supplementary materials can be found at http://www.mdpi.com/1422-0067/19/3/738/s1.

## References

[1] Clevers H. (2013). The intestinal crypt, a prototype stem cell compartment. Cell.

[2] Barker N., van Es J.H., Kuipers J., Kujala P., van den Born M., Cozijnsen M., Haegebarth A., Korving J., Begthel H., Peters P.J. (2007). Identification of stem cells in small intestine and colon by marker gene *Lgr5*. Nature.

[3] De Lau W., Barker N., Low T.Y., Koo B.-K., Li V.S., Teunissen H., Kujala P., Haegebarth A., Peters P.J., van de Wetering M. (2011). Lgr5 homologues associate with Wnt receptors and mediate R-spondin signaling. Nature.

[4] Sato T., Vris R.G., Snippert H.J., van de Wetering M., Barker N., Stange D.E., van Es J.H., Abo A., Kujala P., Peters P.J. (2009). Single Lgr5 stem cells bulid crypt-villus structures in vitro without a mesenchymal niche. Nature.

[5] Grando S.A., Pitterlkow M.R., Schallreuter K.U. (2006). Adrenergic and cholinergic control in the biology of epidermis: Physiological and clinical significance. J. Investig. Dermatol..

[6] Morris D. (1966). The cholinergic acetyltransferase of human placenta. Biochem. J..

[7] Wessler I., Kirkpatrick C.J. (2008). Acetylcholine beyond neurons: The non-neuronal cholinergic system in humans. Br. J. Pharmacol..

[8] Wessler I., Kilbinger H., Bittinger F., Kirkpatrick C.J. (2001). The biological role of non-neuronal acetylcholine in plants and humans. Jpn. J. Pharmacol..

[9] Wessler I., Reinheimer T., Kilbinger F., Bittinger F., Kirkpatrick C.J., Saloga J. (2003). Increased acetylcholine levels in skin biopsies of patients with atopic dermatitis. Life Sci..

[10] Wessler I., Kirkpatrick C.J., Racke K. (1999). The cholinergic ‘pitfall’: Acetylcholine, a universal cell molecule in biological systems, including humans. Clin. Exp. Pharmacol. Physiol..

[11] Clarke A.R. (2006). Wnt signaling in the mouse intestine. Oncogene.

[12] Clevers H. (2006). Wnt/beta-catenin signaling in development and disease. Cell.

[13] Pinto D., Clevers H. (2005). Wnt control of stem cells and differentiation in the intestinal epithelium. Exp. Cell Res..

[14] Cervantes S., Yamaguchi T.P., Hebrok M. (2009). *Wnt5a* is essential for intestinal elongation in mice. Dev. Biol..

[15] Korinek V., Barker N., Moerer P., van Donselaar E., Huls G., Peters P.J., Clevers H. (1998). Depletion of epithelial stem-cell compartments in the small intestine of mice acking Tcf-4. Nat. Genet..

[16] Kim K.A., Zhao J., Andarmani S., Kakitani M., Oshima T., Binnerts M.E., Abo A., Tomizuka K., Funk W.D. (2006). R-spondin proteins: A novel link to beta-catenin activation. Cell Cycle.

[17] Farin H.F., Van Es J.H., Clevers H. (2012). Redundant sources of Wnt regulate intestinal stem cells and promote formation of Paneth cells. Gastroenterology.

[18] Yamaguchi T.P., Bradley A., McMahon A.P., Jones S. (1999). A *Wnt5a* pathway underlies outgrowth of multiple structures in the vertebrate embryo. Development.

[19] Lickert H., kispert A., Kutsch S., Kemler R. (2001). Expression pattern of Wnt genes in mouse gut development. Mech. Dev..

[20] Miyoshi H., Ajima R., Luo C.T., Yamaguchi T.P., Stappenbeck T.S. (2012). *Wnt5a* potentiates TGF-β signaling to promote colonic crypt regeneration after tissue injury. Science.

[21] Takahashi T., Ohnishi H., Sugiura Y., Honda K., Suematsu M., Kawasaki T., Deguchi T., Fujii T., Orihashi K., Hippo Y. (2014). Non-neuronal acetylcholine as an endogenous regulator of proliferation and differentiation of Lgr5-positive stem cells in mice. FEBS J..

[22] Albuquerque E.X., Pereira E.F.R., Alkondon M., Rogers S.W. (2009). Mammalian nicotinic acetylcholine receptors: From structure to function. Physiol. Rev..

[23] Yu Y., Daugherty S., de Groat W.C. (2016). Effects of nicotine receptor agonists on bladder afferent nerve activity in an in vitro bladder-pelvic nerve preparation. Brain Res..

[24] Landgraf D., Barth M., Layer P.G., Sperling L.E. (2010). Acetylcholine as a possible signaling molecule in embryonic stem cells: Studies on survival, proliferation and death. Chem. Biol. Interact..

[25] Ishizuka T., Ozawa A., Goshima H., Watanabe Y. (2012). Involvement of nicotinic acetylcholine receptor in the proliferation of mouse induced plurpotent stem cells. Life Sci..

[26] Cao J., Dwyer J.B., Mangold J.E., Wang J., Wei J., Leslie F.M., Li M.D. (2011). Modulation of cell adhesion systems by prenatal nicotine exposure in limbic brain regions of adolescent female rats. Int. J. Neuropsychopharmacol..

[27] Dennis G.J., Sherman B.T., Hosack D.A., Yang J., Gao W., Lane H.C., Lempicki R.A. (2003). DAVID: Database for Annotation, Visualization, and Integrated Discovery. Genome Biol..

[28] He X., Saint-Jeannet J.P., Wang Y., Nathans J., Dawid I., Varmus H. (1997). A member of the Frizzled protein family mediating axis induction by Wnt-5A. Science.

[29] Mikels A.J., Nusse R. (2006). Purified *Wnt5a* protein activates or inhibits beta-catenin-TCF signaling depending on receptor context. PLoS Biol..

[30] Chen W., ten Berge D., Brown J., Ahn S., Hu L.A., Miller W.E., Caron M.G., Barak L.S., Nusse R., Lefkowitz R.J. (2003). Dishevelled 2 recruits beta-arrestin 2 to mediate *Wnt5a*-stimulated endocytosis of Frizzled 4. Science.

[31] Dejmek J., Sӓfholm A., Nielsen C.K., Andersson T., Leandersson K. (2006). Wnt-5a/Ca^2+^-induced NFAT activity is counteracted by Wnt-5a/Yes-Cdc42-casein kinase 1α signaling in human mammary epithelial cells. Mol. Cell. Biol..

[32] Chen B., Dodge M.E., Tang W., Lu J., Ma Z., Fan C.W., Wei S., Hao W., Kilgore J., Williams N.S. (2009). Small molecule-mediated disruption of Wnt-dependent signaling in tissue regeneration and cancer. Nat. Chem. Biol..

[33] Sato T., van Es J.H., Snippert H.J., Stange D.E., Vries R.G., van den Born M., Barker N., Shroyer N.F., van de Wetering M., Clevers H. (2011). Paneth cells constitute the niche for Lgr5 stem cells in intestinal crypts. Nature.

[34] Conti-Tronconi B.M., McLane K.E., Raftery M.A., Grando S., Protti M.P. (1994). The nicotinic acetylcholine receptor: Structure and auto immune pathology. Crit. Rev. Biochem. Mol. Biol..

[35] Changeux J.P. (1995). Thudichum medal lecture. The acetylcholine receptor: A model for allosteric membrane proteins. Biochem. Soc. Trans..

[36] Lindstrom J.M., North R.A. (1995). Nicotinic acetylcholine receptors. Handbook of Receptors and Channels: Ligand-and Voltage-Gated Ion Channels.

[37] Albuquerque E.X., Alkondon M., Pereira E.F., Castro N.G., Schrattenholz A., Barbosa C.T., Bonfante-Cabarcas R., Aracava Y., Eisenberg H.M., Maelicke A. (1997). Properties of neuronal nicotinic acetylcholine receptors: Pharmacological characterization and modulation of synaptic function. J. Pharmacol. Exp. Ther..

[38] Grando S.A., Horton R.M., Pereira E.F., Diethelm-Okita B.M., Gorge P.M., Albuquerque E.X., Conti-Fine B.M. (1995). A nicotinic acetylcholine receptor regulating cell adhesion and motility is expressed in human keratinocytes. J. Investig. Dermatol..

[39] Conti-Fine B.M., Navaneetham D., Lei S., Maus A.D. (2000). Neuronal nicotinic receptors in non-neuronal cells: New mediators of tobacco toxicity?. Eur. J. Pharmacol..

[40] Wessler I., Kirkpatrick C.J., Racké K. (1998). Non-neuronal acetylcholine, a locally acting molecule, widely distributed in biological systems: Expression and function in humans. Pharmacol. Ther..

[41] Macklin K.D., Maus A.D.J., Pereira E.F.R., Albuquerque E.X., Conti-Fine B.M. (1998). Human vascular endothelial cells express functional nicotinic acetylcholine receptors. J. Pharmacol. Exp. Ther..

[42] Veeman M.T., Axelrod J.D., Moon R.T. (2003). A second canon. Functions and mechanisms of β-catenin-independent Wnt signaling. Dev. Cell.

[43] Kohn A.D., Moon R.T. (2005). Wnt and calcium signaling: β-catenin-independent pathways. Cell Calcium.

[44] Kuhl M., Sheldahl L.C., Park M., Miller J.R., Moon R.T. (2000). The Wnt/Ca^2+^ pathway: A new vertebrate Wnt signaling pathway takes shape. Trends Genet..

[45] Sheldahl L.C., Slusarski D.C., Pandur P., Miller J.R., Kuhl M., Moon R.T. (2003). Dishevelled activates Ca^2+^ flux, PKC, and CamKII in vertebrate embryos. J. Cell Biol..

[46] Tonelli F.M.P., Santos A.K., Gomes D.A., da Silva S.L., Gomes K.N., Ladeira L.O., Resende R.R. (2012). Stem cells and calcium signaling. Adv. Exp. Med. Biol..

[47] Crabtree G.R., Olson E.N. (2002). NFAT signaling: Choreographing the social lives of cells. Cell.

[48] Rao A., Luo C., Hogan P.G. (1997). Transcription factors of the NFAT family: Regulation and function. Annu. Rev. Immunol..

[49] Mo J.S., Park H.W., Guan K.L. (2014). The Hippo signaling pathway in stem cell biology and cancer. EMBO Rep..

[50] Bakker E.R.M., Raghoebir L., Franken P.F., Helvensteijn W., van Gurp L., Meijlink F., van der Valk M.A., Rottier R.J., Kuipers E.J., van Veelen W. (2012). Induced *Wnt5a* expression perturbs embryonic outgrowth and intestinal elongation, but is well-tolerated in adult mice. Dev. Biol..

[51] Cheng C.W., Yeh J.C., Fan T.P., Smith S.K., Charnock-Jones D.S. (2008). *Wnt5a*-mediated non-canonical Wnt signaling regulates human endothelial cell proliferation and migration. Biochem. Biophys. Res. Commun..

[52] Kikuchi A., Yamamoto H., Sato A., Matsumoto S. (2012). *Wnt5a*: Its signaling, functions and implication in disease. Acta. Physiol. (Oxf.).

[53] Nishita M., Enomoto M., Yamagata K., Minami Y. (2010). Cell/tissue-tropic functions of *Wnt5a* signaling in normal and cancer cells. Trends Cell Biol..

[54] Pukrop T., Binder C. (2008). The complex pathways of *Wnt5a* in cancer progression. J. Mol. Med..

[55] Gregorieff A., Pinto D., Begthel H., Destree O., Kielman M., Clevers H. (2005). Expression pattern of Wnt signaling components in the adult intestine. Gastroenterology.

[56] Yamada M., Udagawa J., Matsumoto A., Hashimoto R., Hatta T., Nishita M., Minami Y., Otani H. (2010). Ror2 is required for midgut elongation during mouse development. Dev. Dyn..

[57] Bakker E.R., Das A.M., Helvensteijn W., Franken P.F., Swagemakers S., van der Valk M.A., ten Hagen T.L., Kuipers E.J., van Veelen W., Smits R. (2013). *Wnt5a* promotes human colon cancer cell migration and invasion but does not augment intestinal tumorigenesis in Apc1638N mice. Carcinogenesis.

[58] Cheng R., Sun B., Liu Z., Zhao X., Qi L., Li Y., Gu Q. (2014). *Wnt5a* suppresses colon cancer by inhibiting cell proliferation and epithelial-mesenchymal transition. J. Cell. Physiol..

[59] Posadas I., Lópes-Hernández B., Ceňa V. (2013). Nicotinic receptors in neurodegeneration. Curr. Neuropharmacol..

[60] Eglen R.M., Randle D.H. (2015). Drug discovery goes three-dimensional: Goodbye to flat high-throughput screening?. Assay Drug Dev. Technol..

[61] Martin U. (2015). Pluripotent stem cells for disease modeling and drug screening: New perspectives for treatment of cystic fibrosis?. Mol. Cell. Pediatr..

[62] Sampaziotis F., Cardoso De Brito M., Madrigal P., Bertero A., Saeb-Parsy K., Soares F.A.C., Schrumpf E., Melum E., Karlsen T.H., Bradley J.A. (2015). Cholangiocytes derived from human induced pluripotent stem cells for disease modeling and drug validation. Nat. Biotechnol..

[63] Dedhia P.H., Bertaux-Skeirik N., Zavros Y., Spence J.R. (2016). Organoid models of human gastrointestinal development and disease. Gastroenterology.

[64] Hynds R.E., Giangreco A. (2013). Concise review: The relevance of human stem cell-derived organoids models for epithelial translational medicine. Stem Cells.

[65] Walsh A.J., Cook R.S., Sanders M.E., Arteaga C.L., Skala M.C. (2016). Drug response in organoids generated from frozen primary tumor tissues. Sci. Rep..

