# Supplementary material for: The Coordinated Activities of nAChR and Wnt Signaling Regulate Intestinal Stem Cell Function in Mice
Source: Int J Mol Sci. 2018 Mar 5;19(3):738. doi: 10.3390/ijms19030738 (PMC5877599; doi:10.3390/ijms19030738)
Supplement: Supplementary file 1 [file ijms-19-00738-s001.pdf]

Supplementary

The Coordinated Activities of nAChR and Wnt Signaling Regulate Intestinal Stem Cell Function in Mice

Toshio Takahashi *, Akira Shiraishi and Jun Murata

Suntory Foundation for Life Sciences, Bioorganic Research Institute, Kyoto 619-0284, Japan; shiraishi@sunbor.or.jp (A.S.); murata-j@sunbor.or.jp (J.M.)

* Correspondence: takahashi@sunbor.or.jp; Tel.: +81-50-3182-0707; Fax: +81-774-98-6262

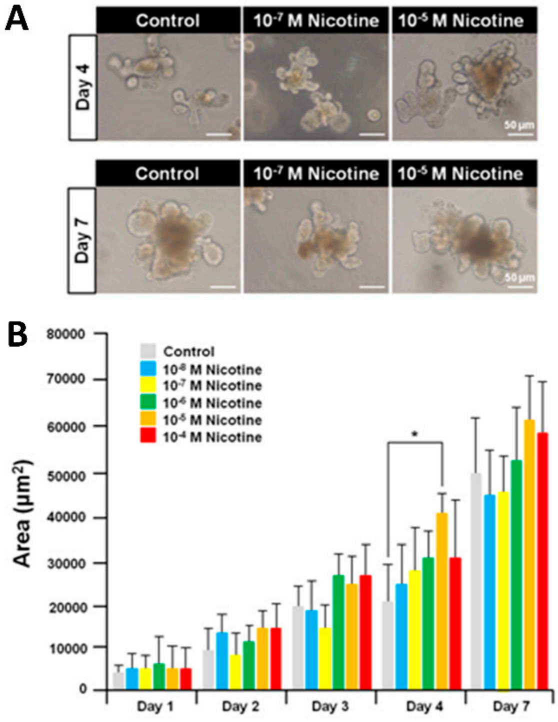

Figure S1. Effect of nicotine on organoid growth. (**A**) Micrographs of organoids after treatment with 10^{-7} M and 10^{-5} M nicotine for 4 days and 7 days of culture. (**B**) Effect of nicotine on the size of cultured organoids. Each sample represents an average of three independent experiments. Error bars represent the standard deviation of the mean. An asterisk indicates a statistically significant difference from untreated control organoids (Mann-Whitney test, $^*p<0.05$).

Table S1. RNA-sequencing analysis of gene expression after 3 days and 7 days of nicotine treatment.

Genbank ID	Gene Name	FPKM[*1]						Fold change[*2]				Pathway
		3 days			7 days			3 days		7 days		
		CTL	10^{-7}M	10^{-5}M	CTL	10^{-7}M	10^{-5}M	10^{-7}M	10^{-5}M	10^{-7}M	10^{-5}M	
NM_001164147	Tcf3	21.36	27.80	26.99	3.04	27.13	90.34	1.30	1.26	8.91	29.67	Stem
NM_011386	Skil	2.10	4.46	6.59	0.64	4.54	7.79	2.13	3.14	7.06	12.13	Stem
NM_021878	Jarid2	2.95	0.00	5.07	2.56	16.75	13.53	0.00	1.71	6.53	5.28	Stem
NM_011548	Tcf3	42.23	40.31	45.10	33.77	63.42	139.21	0.95	1.07	1.88	4.12	Stem
NM_001039537	Lif	5.65	1.51	1.81	1.22	8.72	6.17	0.27	0.32	7.17	5.08	Stem
NM_001164042	Smad5	3.88	0.00	5.80	0.01	2.18	1.77	0.00	1.50	405.58	329.32	Stem
NM_001081149	Kat6a	60.88	63.06	68.05	31.95	72.90	74.69	1.04	1.12	2.28	2.34	Stem
NM_011263	Rest	23.40	25.66	24.51	14.76	36.25	35.08	1.10	1.05	2.46	2.38	Stem
NM_007958	Smarcad1	35.76	45.53	38.51	21.00	38.01	48.28	1.27	1.08	1.81	2.30	Stem
NM_001164153	Tcf3	9.66	4.01	7.26	5.75	11.43	14.01	0.42	0.75	1.99	2.44	Stem
NM_001205043	Jarid2	16.25	15.39	22.60	14.27	22.90	32.82	0.95	1.39	1.60	2.30	Stem
NM_175238	Rif1	52.62	60.79	64.49	39.51	76.49	73.71	1.16	1.23	1.94	1.87	Stem
NM_011535	Tbx3	9.19	23.08	9.13	14.55	25.23	26.73	2.51	0.99	1.73	1.84	Stem
NM_010560	Il6st	24.12	27.08	27.25	15.26	28.89	27.68	1.12	1.13	1.89	1.81	Stem
NM_007496	Zfhx3	8.76	8.75	8.78	6.47	11.87	11.59	1.00	1.00	1.83	1.79	Stem
NM_001163641	Setdb1	2.64	2.60	2.00	4.27	10.00	7.86	0.98	0.76	2.34	1.84	Stem
NM_172716	Pcgf3	88.05	87.14	97.56	57.17	89.48	96.34	0.99	1.11	1.57	1.68	Stem
NM_007395	Acvr1b	141.2	139.4	146.1	112.2	183.0	183.6	0.99	1.03	1.63	1.64	Stem
NM_001077495	Pik3r1	113.9	122.5	124.7	82.1	156.6	149.2	1.08	1.09	1.91	1.82	Stem, THS, Rap1, actin
NM_019827	Gsk3b	74.22	80.95	77.99	55.82	91.30	95.20	1.09	1.05	1.64	1.71	Stem, THS, Hippo, Wnt
NM_007614	Ctnnb1	952.8	864.2	747.4	511.6	824.8	963.8	0.91	0.78	1.61	1.88	Stem, THSHippo, Wnt, Rap1
NM_007561	Bmpr2	23.86	24.02	25.36	18.57	29.62	31.49	1.01	1.06	1.60	1.70	Stem, Hippo
NM_008055	Fzd4	23.10	22.65	21.69	14.33	26.10	28.56	0.98	0.94	1.82	1.99	Stem, Hippo, Wnt
NM_001162494	Fzd6	14.48	14.75	15.65	10.40	20.86	19.53	1.02	1.08	2.01	1.88	Stem, Hippo, Wnt
NM_022721	Fzd5	99.59	104.59	88.47	36.53	67.40	65.63	1.05	0.89	1.84	1.80	Stem, Hippo, Wnt
NM_007889	Dvl3	74.16	79.85	75.89	45.13	78.74	78.07	1.08	1.02	1.74	1.73	Stem, Hippo, Wnt
NM_001256224	Wnt5a	0.15	0.20	0.21	0.00	1.16	0.98	1.30	1.37	4624.5	3885.7	Stem, Hippo, Wnt
NM_011719	Wnt9b	2.43	3.16	3.04	1.82	3.34	2.56	1.30	1.25	1.84	1.41	Stem, Hippo, Wnt
NM_007462	Apc	65.36	69.78	66.21	35.33	68.58	70.15	1.07	1.01	1.94	1.99	Stem, Hippo, Wnt
NM_010207	Fgfr2	15.50	17.67	16.77	15.65	25.61	26.65	1.14	1.08	1.64	1.70	Stem, MAPK, Endoc, Rap1, Actin
NM_001080118	Med1	51.29	56.80	53.88	4.96	58.02	56.25	1.11	1.05	11.70	11.34	THS
NM_012005	Med14	13.53	12.72	11.40	3.89	16.82	17.62	0.94	0.84	4.33	4.53	THS
NM_001205314	Stat1	10.96	3.65	8.51	3.11	4.83	11.42	0.33	0.78	1.55	3.67	THS
NM_001110350	Sin3a	8.08	8.96	12.06	6.11	16.69	19.70	1.11	1.49	2.73	3.22	THS
NM_020005	Kat2b	20.88	14.04	13.74	6.25	15.35	15.95	0.67	0.66	2.45	2.55	THS
NM_008678	Ncoa2	39.88	40.39	41.79	23.07	43.82	52.20	1.01	1.05	1.90	2.26	THS
NM_001252313	Ncor1	124.8	132.4	132.4	77.40	143.1	152.4	1.06	1.06	1.85	1.97	THS
NM_001080931	Med13	58.06	59.16	60.77	30.90	57.87	60.93	1.02	1.05	1.87	1.97	THS
NM_011062	Pdpk1	76.03	87.35	85.04	41.64	77.22	80.73	1.15	1.12	1.85	1.94	THS
NM_001286718	Tsc2	10.21	28.26	24.48	8.81	17.51	16.87	2.77	2.40	1.99	1.92	THS
NM_007503	Fxyd2	0.00	0.26	0.00	0.00	0.52	0.78	264974	1.00	522973	781995	THS
NM_008714	Notch1	46.72	52.65	51.60	30.85	52.23	53.52	1.13	1.10	1.69	1.74	THS
NM_001286713	Tsc2	15.72	11.82	19.20	8.40	14.61	15.02	0.75	1.22	1.74	1.79	THS
NM_172424	Med13l	36.02	32.56	35.60	25.49	47.51	43.44	0.90	0.99	1.86	1.70	THS
NM_021521	Med12	37.89	39.13	38.00	27.09	49.96	46.01	1.03	1.00	1.84	1.70	THS
NM_011308	Ncor1	51.04	56.99	52.30	30.92	54.40	52.07	1.12	1.02	1.76	1.68	THS
NM_008716	Notch3	0.91	1.35	1.08	3.14	5.47	5.81	1.49	1.20	1.74	1.85	THS
NM_177229	Ncor1	12.77	16.96	17.19	20.51	31.96	34.34	1.33	1.35	1.56	1.67	THS
NM_011378	Sin3a	11.46	12.63	4.57	4.34	7.40	7.66	1.10	0.40	1.70	1.76	THS
NM_001025432	Crebbp	37.65	37.26	36.89	17.36	38.44	37.54	0.99	0.98	2.21	2.16	THS, Wnt
NM_177821	Ep300	76.51	76.01	75.87	41.31	80.85	79.91	0.99	0.99	1.96	1.93	THS, Wnt
NM_001145830	Plcb1	0.00	0.07	2.02	0.00	1.97	3.52	359.67	10805.6	4295.0	7695.3	THS, Wnt, Rap1
NM_001005784	Patj	17.59	6.81	10.91	2.81	10.49	10.89	0.39	0.62	3.74	3.88	Hippo
NM_026735	Mob1b	98.07	112.8	107.5	43.32	98.40	102.9	1.15	1.10	2.27	2.37	Hippo
NM_007862	Dlg1	36.66	43.47	43.25	37.47	65.31	73.37	1.19	1.18	1.74	1.96	Hippo
NM_001252253	Nf2	0.00	1.77	0.86	0.73	2.23	2.27	1770261	856702	3.07	3.11	Hippo
NM_001109752	Dlg4	0.04	0.68	0.30	1.07	2.44	2.80	16.37	7.20	2.28	2.62	Hippo
NM_145571	Mob1a	261.5	272.1	273.8	179.4	295.0	330.2	1.04	1.05	1.64	1.84	Hippo
NM_001166585	Tead1	18.50	30.61	24.13	19.68	38.42	32.32	1.65	1.30	1.95	1.64	Hippo
NM_010690	Lats1	81.69	90.21	88.21	57.88	94.78	93.62	1.10	1.08	1.64	1.62	Hippo
NM_134015	Fbxw11	0.00	2.60	3.39	0.00	5.80	4.56	1819.1	2373.5	11826.2	9287.3	Hippo, Wnt
NM_001271347	Fbxw11	1.62	1.69	0.01	1.23	3.25	5.18	1.04	0.01	2.65	4.22	Hippo, Wnt
NM_001271348	Fbxw11	53.57	56.79	55.64	32.31	67.58	64.46	1.06	1.04	2.09	2.00	Hippo, Wnt
NM_001142920	Tcf7l2	19.60	24.13	24.20	13.06	24.64	23.58	1.23	1.23	1.89	1.81	Hippo, Wnt
NM_009371	Tgfbr2	68.42	45.51	62.30	30.71	101.4	96.22	0.67	0.91	3.30	3.13	Hippo, MAPK, Endoc
NM_001122850	Pard3	36.14	34.58	37.05	29.69	48.20	49.14	0.96	1.03	1.62	1.66	Hippo, Endocytosis, Rap1
NM_020601	Tbl1x	129.4	140.4	140.8	104.8	216.3	226.6	1.08	1.09	2.06	2.16	Wnt

NM_001004367	Cxxc4	0.52	0.81	0.60	0.46	1.22	1.58	1.56	1.16	2.63	3.41	Wnt
NM_008514	Lrp6	70.38	74.94	78.31	47.65	80.35	82.44	1.06	1.11	1.69	1.73	Wnt
NM_013502	Ctbp1	40.62	41.05	44.38	25.53	42.38	42.60	1.01	1.09	1.66	1.67	Wnt
NM_172688	Map3k7	2.63	1.81	1.62	0.00	2.17	3.04	0.69	0.62	17479.4	24452.2	Wnt, MAPK
NM_001164112	Nfatc1	4.49	5.37	3.91	2.95	4.71	5.86	1.20	0.87	1.60	1.99	Wnt, MAPK
NM_013931	Mapk8ip3	1.66	13.56	9.65	0.00	6.35	11.29	8.18	5.82	5197650	9245016	MAPK
NM_001163453	Mapk8ip3	32.12	23.92	30.11	4.59	43.76	37.46	0.74	0.94	9.54	8.16	MAPK
NM_008696	Map4k4	6.43	4.14	7.18	0.00	1.97	5.78	0.64	1.12	446.54	1310.89	MAPK
NM_001285488	Mknk1	0.00	0.00	0.00	0.00	2.36	3.89	0.90	1.34	7221.68	11869.48	MAPK
NM_001163643	Map3k12	1.03	0.00	1.04	0.00	1.09	2.89	0.00	1.01	3543.67	9417.03	MAPK
NM_009784	Cacna2d1	1.36	2.25	1.86	0.01	2.21	2.61	1.65	1.36	350.92	414.06	MAPK
NM_007923	Elk4	36.98	38.84	41.78	12.93	43.41	40.98	1.05	1.13	3.36	3.17	MAPK
NM_001038609	Mapt	2.22	0.00	2.23	1.00	3.33	3.69	0.00	1.00	3.33	3.68	MAPK
NM_148945	Rps6ka3	101.4	101.2	108.2	56.6	132.9	128.8	1.00	1.07	2.35	2.27	MAPK
NM_172821	Map3k13	20.32	21.87	23.39	8.59	19.08	19.42	1.08	1.15	2.22	2.26	MAPK
NM_001161423	Ikbkg	0.72	1.08	0.00	0.00	0.23	1.07	1.50	0.00	690.35	3220.9	MAPK
NM_001252060	Cacna1a	1.54	3.32	7.09	1.68	7.86	4.47	2.16	4.61	4.67	2.65	MAPK
NM_001284371	Atf2	2.68	3.62	2.82	1.53	2.85	3.76	1.35	1.05	1.86	2.45	MAPK
NM_144825	Taok1	41.52	45.14	46.44	26.07	48.80	48.77	1.09	1.12	1.87	1.87	MAPK
NM_001252202	Map4k4	20.13	23.46	29.01	24.23	44.84	45.25	1.16	1.44	1.85	1.87	MAPK
NM_007746	Map3k8	1.80	1.55	2.31	1.39	2.96	3.34	0.86	1.28	2.14	2.41	MAPK
NM_013560	Hspb1	1.04	0.28	0.28	0.00	0.27	0.77	0.27	0.27	267089	767023	MAPK
NM_134080	Flnb	158.8	158.3	160.4	127.8	202.0	222.0	1.00	1.01	1.58	1.74	MAPK
NM_010897	Nf1	23.12	24.34	23.48	17.16	29.67	30.39	1.05	1.02	1.73	1.77	MAPK
NM_011946	Map3k2	39.78	40.79	43.89	21.75	37.74	36.97	1.03	1.10	1.74	1.70	MAPK
NM_001174049	Cacna2d2	0.20	0.19	0.11	0.72	1.46	1.83	0.94	0.53	2.02	2.53	MAPK
NM_001025093	Atf2	23.47	24.38	22.69	12.38	32.70	20.71	1.04	0.97	2.64	1.67	MAPK
NM_008301	Hspa2	2.23	2.17	2.70	0.00	4.03	3.70	0.97	1.21	1853.2	1702.1	MAPK, Endoc
NM_007764	Crkl	64.19	75.31	70.71	44.28	73.64	73.83	1.17	1.10	1.66	1.67	MAPK, Rap1, Actin
NM_139294	Braf	17.69	20.93	20.70	16.86	27.69	28.10	1.18	1.17	1.64	1.67	MAPK, Rap1, Actin
NM_001177558	Gng12	31.82	18.23	30.57	8.08	23.46	24.62	0.57	0.96	2.90	3.05	MAPK, Actin
NM_001162868	Rab11fip3	9.01	0.03	15.19	0.01	4.50	12.85	0.00	1.69	440.48	1258.2	Endocytosis
NM_007619	Cbl	42.11	44.01	45.33	9.18	44.98	50.96	1.05	1.08	4.90	5.55	Endocytosis
NM_001134383	Iqsec1	7.51	5.68	6.88	1.92	9.22	11.27	0.76	0.92	4.81	5.87	Endocytosis
NM_001276462	Asap1	0.00	4.21	0.40	1.08	3.38	5.32	1756.6	166.1	3.14	4.94	Endocytosis
NM_001164056	Pld1	12.30	10.84	6.85	8.05	13.95	19.38	0.88	0.56	1.73	2.41	Endocytosis
NM_029438	Smurf1	4.03	9.39	6.64	6.46	11.20	15.75	2.33	1.65	1.73	2.44	Endocytosis
NM_001080813	Rab11fip1	35.82	40.67	36.44	18.07	31.64	37.77	1.14	1.02	1.75	2.09	Endocytosis
NM_001164678	Pdcd6ip	10.32	16.65	20.95	9.91	18.52	20.88	1.61	2.03	1.87	2.11	Endocytosis
NM_010153	Erbb3	219.6	223.5	233.1	106.7	208.8	214.5	1.02	1.06	1.96	2.01	Endocytosis
NM_001114664	Iqsec2	4.94	7.85	7.72	4.04	7.19	8.94	1.59	1.56	1.78	2.21	Endocytosis
NM_001144988	Spg20	17.52	12.64	14.73	4.51	12.59	9.73	0.72	0.84	2.79	2.16	Endocytosis
NM_001285806	Grk3	3.41	0.00	1.93	4.08	10.02	8.88	0.00	0.56	2.45	2.17	Endocytosis
NM_001164367	Rab11fip2	3.45	3.63	3.09	2.69	6.88	5.98	1.05	0.90	2.55	2.22	Endocytosis
NM_027487	Ccdc53	2.23	4.93	5.82	3.53	6.28	7.10	2.21	2.61	1.78	2.01	Endocytosis
NM_030263	Psd3	10.91	14.83	14.77	9.74	20.75	17.92	1.36	1.35	2.13	1.84	Endocytosis
NM_173392	Zfyve16	14.55	15.77	15.70	13.84	23.96	24.65	1.08	1.08	1.73	1.78	Endocytosis
NM_001085495	Arfgef2	81.76	87.55	87.71	43.69	72.39	75.94	1.07	1.07	1.66	1.74	Endocytosis
NM_025519	Chmp4c	142.7	141.0	169.0	106.80	171.1	182.4	0.99	1.18	1.60	1.71	Endocytosis
NM_175543	Rab11fip4	2.83	2.38	3.41	3.65	6.81	6.83	0.84	1.21	1.87	1.87	Endocytosis
NM_001122832	Eps15l1	43.08	43.79	42.06	27.14	42.39	45.29	1.02	0.98	1.56	1.67	Endocytosis
NM_001040111	Arap1	30.20	26.74	28.65	16.39	25.83	27.38	0.89	0.95	1.58	1.67	Endocytosis
NM_001033375	A230046	47.18	50.63	52.02	34.17	53.32	56.10	1.07	1.10	1.56	1.64	Endocytosis
NM_010515	Igf2r	83.47	89.92	88.18	63.01	102.43	102.81	1.08	1.06	1.63	1.63	Endocytosis
NM_001122733	Kit	7.64	7.51	10.68	4.01	16.48	14.59	0.98	1.40	4.11	3.64	Endoc, Rap1
NM_001167745	Wasl	1.46	2.42	2.61	0.66	1.82	2.66	1.66	1.79	2.77	4.03	Endoc, Actin
NM_001286785	Magi1	2.66	0.00	4.29	0.00	5.87	4.04	0.00	1.61	22016.1	15153.3	Rap1
NM_001167983	Sipa1l1	17.00	20.16	17.18	9.07	31.30	40.89	1.19	1.01	3.45	4.51	Rap1
NM_001162425	Efna1	1.11	1.13	2.20	0.00	1.18	2.25	1.02	1.99	11176.7	21339.5	Rap1
NM_178900	Prkd2	2.16	2.68	5.14	0.53	3.69	3.55	1.24	2.37	6.97	6.70	Rap1
NM_001204165	Rapgef4	0.00	0.00	0.00	0.00	2.59	1.64	91.70	403.31	2881.4	1824.2	Rap1
NM_001171004	Prkd3	20.24	24.80	26.47	14.58	41.91	37.37	1.23	1.31	2.87	2.56	Rap1
NM_001145834	Ralgds	10.22	0.00	15.63	8.96	27.76	22.40	0.00	1.53	3.10	2.50	Rap1
NM_175930	Rapgef5	15.41	14.72	15.09	9.90	21.57	24.16	0.95	0.98	2.18	2.44	Rap1
NM_009624	Adcy9	15.19	13.48	14.21	7.09	12.95	16.23	0.89	0.94	1.83	2.29	Rap1
NM_172803	Dock4	4.70	6.21	6.53	3.06	7.27	7.57	1.32	1.39	2.38	2.48	Rap1
NM_001085450	Ctnnd1	23.76	41.94	29.08	27.28	43.09	55.86	1.77	1.22	1.58	2.05	Rap1
NM_001163269	Lpar5	70.77	80.03	82.57	33.98	55.19	64.75	1.13	1.17	1.62	1.91	Rap1
NM_001286784	Magi1	22.79	21.12	24.94	14.32	27.85	25.98	0.93	1.09	1.95	1.81	Rap1
NM_001159354	Magi3	62.99	69.44	62.65	39.27	67.84	69.86	1.10	0.99	1.73	1.78	Rap1
NM_175258	Rapgef6	39.78	43.44	42.49	26.70	45.14	45.61	1.09	1.07	1.69	1.71	Rap1
NM_009623	Adcy8	9.17	11.75	9.43	5.17	7.95	9.07	1.28	1.03	1.54	1.76	Rap1
NM_001145886	Tiam1	27.91	30.38	33.07	10.63	36.10	45.54	1.09	1.19	3.39	4.28	Rap1, Actin
NM_001082960	Itgam	5.05	5.61	4.97	0.00	2.76	1.33	1.11	0.98	1350.3	650.99	Rap1, Actin
NM_001130409	Ptk2	12.68	11.15	10.93	4.89	13.38	17.31	0.88	0.86	2.74	3.54	Actin

NM_001081307	Ppp1r12b	8.37	8.95	8.29	2.09	7.17	7.45	1.07	0.99	3.44	3.57	Actin
NM_008396	Itga2	35.99	38.37	39.36	26.60	63.55	69.46	1.07	1.09	2.39	2.61	Actin
NM_001005608	Itgb4	0.69	6.09	3.09	1.84	4.62	4.79	8.86	4.49	2.52	2.61	Actin
NM_001033228	Itga1	16.08	17.78	17.15	16.27	30.11	31.77	1.11	1.07	1.85	1.95	Actin
NM_172493	Diaph2	11.13	11.55	10.16	9.96	19.11	19.42	1.04	0.91	1.92	1.95	Actin
NM_027892	Ppp1r12a	75.89	82.69	78.50	54.66	96.74	102.46	1.09	1.03	1.77	1.87	Actin
NM_027144	Arhgef12	66.62	72.52	70.94	45.73	89.33	84.88	1.09	1.06	1.95	1.86	Actin
NM_198127	Abi2	0.60	1.08	0.98	0.19	1.41	1.16	1.80	1.63	7.28	5.99	Actin
NM_153423	Wasf2	130.8	145.1	135.4	85.11	150.2	152.1	1.11	1.03	1.76	1.79	Actin
NM_001113517	Arhgef7	1.88	3.07	1.42	1.34	3.43	3.16	1.63	0.76	2.55	2.35	Actin
NM_177710	Ssh2	29.89	33.83	33.78	19.91	32.84	33.83	1.13	1.13	1.65	1.70	Actin
NM_015759	Fgd3	0.97	0.78	1.47	1.35	2.34	2.85	0.80	1.52	1.74	2.12	Actin

[*1]The FPKM (Fragments Per Kilobase Mega read) values. CTL, Control.
[*2]Fold change is defined as the ratio of the signal of non-treated organoids to that of treated organoids.

Table S2. RNA-sequencing analysis of gene expression after 7 days of mecamylamine treatment.

Genbank ID	Gene Name	FPKM[*1]		Fold change[*2]	Pathway
		CTL	10^{-6} M	10^{-6} M	
NM_001164147	Tcf3	2.57	1.52	0.59	Stem
NM_011386	Skil	0.00	1.57	15909.89	Stem
NM_021878	Jarid2	1.14	1.10	0.97	Stem
NM_011548	Tcf3	5.30	6.24	1.18	Stem
NM_001039537	Lif	1.59	1.33	0.84	Stem
NM_001164042	Smad5	0.00	0.00	0.53	Stem
NM_001081149	Kat6a	8.90	9.03	1.01	Stem
NM_011263	Rest	3.66	3.46	0.95	Stem
NM_007958	Smarcad1	5.80	5.43	0.94	Stem
NM_001164153	Tcf3	0.37	0.00	0.00	Stem
NM_001205043	Jarid2	2.19	1.17	0.54	Stem
NM_175238	Rif1	9.67	9.57	0.99	Stem
NM_011535	Tbx3	1.89	0.07	0.04	Stem
NM_010560	Il6st	3.72	4.29	1.15	Stem
NM_007496	Zfhx3	1.28	1.11	0.87	Stem
NM_001163641	Setdb1	1.13	0.00	0.00	Stem
NM_172716	Pcgf3	11.91	10.89	0.91	Stem
NM_007395	Acvr1b	21.70	19.61	0.90	Stem
NM_001077495	Pik3r1	15.54	15.35	0.99	Stem, THS, Rap1, actin
NM_019827	Gsk3b	10.01	9.98	1.00	Stem, THS, Hippo, Wnt
NM_007614	Ctnnb1	81.21	68.20	0.84	Stem, THSHippo, Wnt, Rap1
NM_007561	Bmpr2	3.89	3.82	0.98	Stem, Hippo
NM_008055	Fzd4	3.82	3.16	0.83	Stem, Hippo, Wnt
NM_001162494	Fzd6	1.62	1.65	1.02	Stem, Hippo, Wnt
NM_022721	Fzd5	7.96	7.76	0.97	Stem, Hippo, Wnt
NM_007889	Dvl3	8.24	9.05	1.10	Stem, Hippo, Wnt
NM_001256224	Wnt5a	0.02	0.00	0.00	Stem, Hippo, Wnt
NM_011719	Wnt9b	0.05	0.18	0.29	Stem, Hippo, Wnt
NM_007462	Apc	8.54	8.23	0.96	Stem, Hippo, Wnt
NM_010207	Fgfr2	1.92	2.39	1.25	Stem, MAPK, Endoc, Rap1, Actin
NM_001080118	Med1	9.18	8.78	0.96	THS
NM_012005	Med14	2.57	4.12	1.60	THS
NM_001205314	Stat1	1.48	1.24	0.84	THS
NM_001110350	Sin3a	3.96	1.69	0.43	THS
NM_020005	Kat2b	1.10	0.41	0.37	THS
NM_008678	Ncoa2	4.81	6.23	1.30	THS
NM_001252313	Ncor1	18.51	18.04	0.97	THS
NM_001080931	Med13	6.31	6.22	0.98	THS
NM_011062	Pdpk1	9.16	7.76	0.85	THS
NM_001286718	Tsc2	0.92	1.05	1.15	THS
NM_007503	Fxyd2	0.00	0.00	1.00	THS
NM_008714	Notch1	4.93	4.90	0.99	THS
NM_001286713	Tsc2	0.00	1.76	1577.57	THS
NM_172424	Med13l	4.90	5.19	1.06	THS
NM_021521	Med12	6.45	6.73	1.04	THS
NM_011308	Ncor1	6.61	8.16	1.24	THS
NM_008716	Notch3	0.51	0.36	0.71	THS
NM_177229	Ncor1	3.83	2.86	0.75	THS

NM_011378	Sin3a	1.61	1.10	0.68	THS
NM_001025432	Crebbp	3.83	3.80	0.99	THS, Wnt
NM_177821	Ep300	8.84	8.71	0.99	THS, Wnt
NM_001145830	Plcb1	0.32	0.02	0.07	THS, Wnt, Rap1
NM_001005784	Patj	0.76	0.81	1.06	Hippo
NM_026735	Mob1b	11.70	9.75	0.83	Hippo
NM_007862	Dlg1	5.16	8.55	1.66	Hippo
NM_001252253	Nf2	0.00	0.40	397838.81	Hippo
NM_001109752	Dlg4	0.00	0.00	0.80	Hippo
NM_145571	Mob1a	38.46	37.04	0.96	Hippo
NM_001166585	Tead1	4.36	4.91	1.13	Hippo
NM_010690	Lats1	11.01	10.81	0.98	Hippo
NM_134015	Fbxw11	0.71	0.00	0.00	Hippo, Wnt
NM_001271347	Fbxw11	0.63	0.00	0.00	Hippo, Wnt
NM_001271348	Fbxw11	8.64	7.20	0.83	Hippo, Wnt
NM_001142920	Tcf7l2	2.07	2.34	1.13	Hippo, Wnt
NM_009371	Tgfbr2	4.01	7.30	1.82	Hippo, MAPK, Endoc
NM_001122850	Pard3	5.91	4.94	0.84	Hippo, Endocytosis, Rap1
NM_020601	Tbl1x	30.33	27.33	0.90	Wnt
NM_001004367	Cxxc4	0.05	0.15	3.04	Wnt
NM_008514	Lrp6	9.12	9.16	1.00	Wnt
NM_013502	Ctbp1	3.20	2.36	0.74	Wnt
NM_172688	Map3k7	0.58	0.53	0.91	Wnt, MAPK
NM_001164112	Nfatc1	0.48	0.00	0.00	Wnt, MAPK
NM_013931	Mapk8ip3	1.54	0.42	0.27	MAPK
NM_001163453	Mapk8ip3	4.57	2.47	0.54	MAPK
NM_008696	Map4k4	1.11	0.00	0.00	MAPK
NM_001285488	Mknk1	0.00	0.00	6.28	MAPK
NM_001163643	Map3k12	0.50	0.00	0.00	MAPK
NM_009784	Cacna2d1	0.21	0.39	1.84	MAPK
NM_007923	Elk4	6.27	6.61	1.05	MAPK
NM_001038609	Mapt	0.00	0.00	1.16	MAPK
NM_148945	Rps6ka3	15.01	15.05	1.00	MAPK
NM_172821	Map3k13	2.66	2.98	1.12	MAPK
NM_001161423	Ikbkg	0.43	0.00	0.00	MAPK
NM_001252060	Cacna1a	1.09	0.99	0.91	MAPK
NM_001284371	Atf2	0.66	0.34	0.52	MAPK
NM_144825	Taok1	6.16	6.33	1.03	MAPK
NM_001252202	Map4k4	3.28	2.72	0.83	MAPK
NM_007746	Map3k8	0.75	1.02	1.37	MAPK
NM_013560	Hspb1	0.12	0.00	0.00	MAPK
NM_134080	Flnb	30.98	27.32	0.88	MAPK
NM_010897	Nf1	3.89	3.75	0.97	MAPK
NM_011946	Map3k2	4.29	4.15	0.97	MAPK
NM_001174049	Cacna2d2	0.36	0.17	0.48	MAPK
NM_001025093	Atf2	3.29	2.69	0.82	MAPK
NM_008301	Hspa2	0.65	0.37	0.57	MAPK, Endoc
NM_007764	Crkl	8.08	7.49	0.93	MAPK, Rap1, Actin
NM_139294	Braf	3.60	3.45	0.96	MAPK, Rap1, Actin
NM_001177558	Gng12	0.96	0.00	0.00	MAPK, Actin
NM_001162868	Rab11fip3	1.32	0.00	0.00	Endocytosis
NM_007619	Cbl	7.00	7.29	1.04	Endocytosis
NM_001134383	Iqsec1	1.18	0.27	0.23	Endocytosis
NM_001276462	Asap1	0.71	0.00	0.00	Endocytosis
NM_001164056	Pld1	3.60	0.00	0.00	Endocytosis
NM_029438	Smurf1	0.94	1.43	1.51	Endocytosis
NM_001080813	Rab11fip1	6.24	5.41	0.87	Endocytosis
NM_001164678	Pdcd6ip	1.29	1.95	1.51	Endocytosis
NM_010153	Erbb3	25.80	25.04	0.97	Endocytosis
NM_001114664	Iqsec2	0.79	0.93	1.18	Endocytosis
NM_001144988	Spg20	0.00	1.07	14798.36	Endocytosis
NM_001285806	Grk3	0.00	0.00	2.25	Endocytosis
NM_001164367	Rab11fip2	0.39	0.61	1.59	Endocytosis
NM_027487	Ccdc53	0.00	1.33	102631.60	Endocytosis
NM_030263	Psd3	2.60	2.45	0.94	Endocytosis
NM_173392	Zfyve16	3.00	2.85	0.95	Endocytosis
NM_001085495	Arfgef2	10.30	9.15	0.89	Endocytosis
NM_025519	Chmp4c	22.60	23.80	1.05	Endocytosis
NM_175543	Rab11fip4	0.70	0.31	0.43	Endocytosis
NM_001122832	Eps15l1	6.59	5.20	0.79	Endocytosis
NM_001040111	Arap1	3.98	2.39	0.60	Endocytosis
NM_001033375	A230046K03Rik	10.67	10.12	0.95	Endocytosis
NM_010515	Igf2r	10.45	9.27	0.89	Endocytosis
NM_001122733	Kit	2.58	1.13	0.44	Endoc, Rap1
NM_001167745	Wasl	0.51	0.33	0.65	Endoc, Actin
NM_001286785	Magi1	0.00	0.00	0.12	Rap1
NM_001167983	Sipa1l1	4.58	3.27	0.71	Rap1
NM_001162425	Efna1	0.00	0.00	1.32	Rap1
NM_178900	Prkd2	1.19	0.69	0.58	Rap1
NM_001204165	Rapgef4	0.00	0.00	0.02	Rap1

NM_001171004	Prkd3	4.85	5.81	1.20	Rap1
NM_001145834	Ralgds	3.25	3.41	1.05	Rap1
NM_175930	Rapgef5	3.54	3.39	0.96	Rap1
NM_009624	Adcy9	1.87	1.22	0.65	Rap1
NM_172803	Dock4	0.73	1.00	1.38	Rap1
NM_001085450	Ctnnd1	4.76	3.79	0.80	Rap1
NM_001163269	Lpar5	5.87	2.14	0.36	Rap1
NM_001286784	Magi1	3.84	1.87	0.49	Rap1
NM_001159354	Magi3	6.04	5.26	0.87	Rap1
NM_175258	Rapgef6	4.91	5.11	1.04	Rap1
NM_009623	Adcy8	0.23	0.33	1.40	Rap1
NM_001145886	Tiam1	5.91	1.88	0.32	Rap1, Actin
NM_001082960	Itgam	1.41	0.59	0.42	Rap1, Actin
NM_001130409	Ptk2	1.83	3.65	1.99	Actin
NM_001081307	Ppp1r12b	0.82	1.07	1.30	Actin
NM_008396	Itga2	8.54	7.42	0.87	Actin
NM_001005608	Itgb4	0.14	0.55	3.96	Actin
NM_001033228	Itga1	2.26	3.02	1.34	Actin
NM_172493	Diaph2	2.22	2.62	1.18	Actin
NM_027892	Ppp1r12a	18.43	19.40	1.05	Actin
NM_027144	Arhgef12	11.42	11.48	1.00	Actin
NM_198127	Abi2	0.42	0.32	0.77	Actin
NM_153423	Wasf2	20.78	19.01	0.92	Actin
NM_001113517	Arhgef7	0.21	0.22	1.05	Actin
NM_177710	Ssh2	3.59	3.91	1.09	Actin
NM_015759	Fgd3	0.50	0.27	0.54	Actin

*1The FPKM (Fragments Per Kilobase Mega read) values. CTL, Control.
*2Fold change is defined as the ratio of the signal of non-treated organoids to that of treated organoids.

Table S3. Primers for RT-PCR.

Gene	Forward primer	Reverse primer
α1	TCCCTTCGATGAGCAGAACT	ATGATGGACGCAATGACAAA
α2	TCCCTCACTGTCTTCCTGCT	ACACCCATGGAAGAGTCTGG
α3	CCTTCCACCTCACTGGTCAT	CAGAGCAGACAGGGACAACA
α4	CCGGAGACTTATCGAATCCA	TGGTCTGACACTGGAAGCTG
α5	AAAAGTGGGTTCGTCCTGTG	ATGAGCCGAATTTCATGGAG
α6	AACCTGCACTCCGGTTTATG	TCCAGGTTATCACACCGTCA
α7	CCCGGAGTGAAAAATGTTCGT	TGAGCACAAGGAATGAGC
α9	GATCGATGGGCTCATCACCT	AACGATATGAGGACGCAAGG
α10	CGTTCCACTCATCGGAAAGT	GGCAATGGAAGCTACGTGAT
β1	GGAGAGGAGAGGCAGGAAGT	GGAGAAGGCGACAAGGACCA
β2	TTTGACCAGCAGAACTGCAC	GTACTTTCCCACCAGCGGTA
β3	CGATGGAATCCCGAAGACTA	CAGGCAGGGGATTATCAAAA
β4	TTCCATCGTCACCACTGTGT	TCACTCTCCAAATGCTGTGC
Lgr5	GTCTCCTACATCGCCTCTGC	TTCCAAAGGCGTAGTCTGCT
Sox9	AGGAAGCTGGCAGACCAGTA	CCCTCTCGCTTCAGATCAAC
Ngn3	CCCCAGAGACACAACAACCT	TCTGAGTCAGTGCCCAGATG
Hnf1	GAGAGGTGGCTCAGCAATTC	GCCGCAGACACTGTGACTAA
Klf4	CAGCTTCAGCTATCCGATCC	CGCCTCTTGCTTAATCTTGG
GAPDH	AACTTTGGCATTGTGGAAGG	ACACATTGGGGGTAGGAACA

Table S4. Primers for quantitative RT-PCR.

Gene	Forward primer	Reverse primer
Lgr5	AGCATACCCGTTTCTGGATG	AGGACCGTTTCTCAACATCG
Sox9	ATAAGTTCCCCGTGTGCATC	TGACGTGTGGCTTGTTCTTG
Ngn3	AAGCAGAAGTGGGTGACTGC	TCTTCGCTGTTTGCTGAGTG
Hnf1	AAAACCCCAGCAAGGAAGAG	GGTTGGCAAACCAGTTGTAG
Klf4	AGCATACCCGTTTCTGGATG	CTGTGTGTTTGCGGTAGTGCC
Wnt5a	CAAATAGGCAGCCGAGAGAC	CTCTAGCGTCCACGAACTCC
α2	ATCGTGCGCTTTGGACTATC	TTAGCCAGACATTGGTGGTC
β4	TGCTGGCACTCACATTCTTC	AGTGGTGACGATGGAAAAGG
GAPDH	TGACGTGCCGCCTGGAGAAA	AGTGTAGCCCAAGATGCCCTTCAG
